# Dissection and Engineering of Modular Polyketide Synthase Extender Unit Specificity Motifs

**DOI:** 10.1002/cbic.70447

**Published:** 2026-07-10

**Authors:** Sydney Welch, Gavin J. Williams

**Affiliations:** ^1^ Department of Chemistry NC State University Raleigh North Carolina USA; ^2^ Comparative Medicine Institute NC State University Raleigh North Carolina USA

**Keywords:** acyl‐CoA, enzymes, polyketide synthase, polyketides, substrate specificity

## Abstract

Polyketide synthases (PKSs) are privileged enzymatic platforms for generating natural products. Yet, efforts to redesign their acyltransferase (AT) domains for incorporation of non‐native extender units remain constrained by an incomplete understanding of the sequence elements that govern specificity. EryAT6 is the methylmalonyl‐CoA‐specific terminal AT domain of the erythromycin PKS (DEBS). Two conserved motifs within EryAT6, the large‐ and small‐subunit motifs (LSM and SSM), suggest that short sequence segments encode key extender unit selectivity, but their modularity has not been systematically explored. Here, we delineate the sequence–function relationships underlying extender unit selection by combining mutagenesis, motif swapping, and functional reconstitution in Ery6TE. Substitution of ten nonconserved residues across the LSM and SSM revealed some positions that strongly influence the incorporation of larger extender units. Triple‐residue combinations exhibited cooperativity, with the T739A/Y744R/S746G mutant increasing the portion of butylmalonyl‐CoA‐derived product by 42‐fold. Motif exchanges showed that both LSM and SSM can reprogram AT selectivity in AT‐swapped Ery6TE chimeras, enabling the formation of butyl‐substituted pyrones even when the parent domain swap was inactive. Together, these results identify compact determinants of extender unit specificity and establish motif‐level engineering as a strategy to access diverse polyketides.

## Graphical abstract

Motif‐level engineering of acyltransferase domains in polyketide synthases enables programable extender unit selectivity. Cooperative mutations and motif exchanges reprogram substrate preference, enabling the incorporation of non‐native extender units and expanding polyketide structural diversity, with gains in activity and product yield.

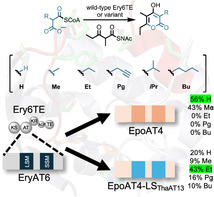

## 1 Introduction

**FIGURE 1:**
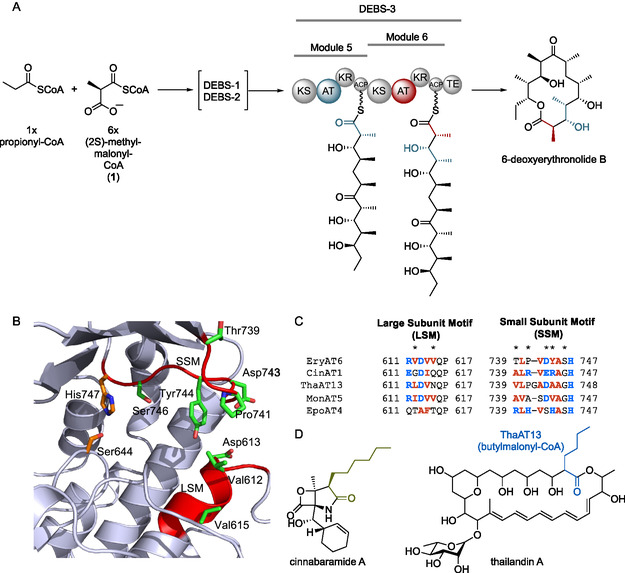
Type I PKS organization and extender unit specificity motifs. (A) Scheme showing the last two modules of the 6‐deoxyerythronolide B synthase (DEBS). (B) Model of the EryAT6 from DEBS. The LSM and SSM are shown in red. Selected side chains of residues in the YASH, LSM, and SSM motifs are shown in green, in addition to catalytic residues in orange (Ser644, His747). (C) LSM and SSM sequences in EryAT6, CinAT1, and ThaAT13. (D) Structures of cinnabaramide A and thailandin A and the role of CinAT1 and ThaAT13.

Polyketides have been represented in some of the most critical drug discoveries in the last 70 years [1]. Yet, engineering the biosynthesis of new derivatives remains challenging and cannot always compete with even lengthy, low‐yielding synthetic routes [2]. The biosynthesis of polyketide scaffolds is catalyzed by large enzyme complexes called polyketide synthases (PKSs). Polyketide biosynthesis begins with small carbon building blocks, called extender units, which are connected via a decarboxylative thio‐Claisen condensation to form a polyketide chain [3]. In type I polyketides, such as erythromycin A (ErA), biosynthesis occurs via an assembly line in which the multidomain enzymes are arranged in a linear fashion and promote polyketide chain extension stepwise (Figure 1A) [4]. For instance, the biosynthetic gene cluster for ErA encodes the machinery for three large multifunctional polypeptides, each organized into two modules. Each module requires a minimum of three enzymes per single‐chain extension: the acyl carrier protein (ACP), the acyltransferase (AT), and the ketosynthase (KS). The ACP serves to covalently carry the extender unit (**1**) or intermediates. In the holo (active) form of the enzyme, a phosphopantetheine arm forms a thioester linkage with the extender unit through a serine residue, which then passes the extender unit downstream to the KS. ATs act as filters for which extender units are loaded onto the ACP—they are highly specific for their cognate extenders, as there often are multiple competing within a cell. An extender unit recognized by the AT forms an acyl‐*O*‐AT intermediate and then passes the extender unit to the ACP phosphopantetheine arm [5]. In the last elongation step, the KS catalyzes the condensation of the acceptor and extender units. Beyond the minimally required enzymes, each module may contain additional tailoring enzymes, such as a ketoreductase (KR), dehydratase (DH), methyltransferase (MT), and enoyl reductase (ER).

Malonyl‐CoA, methylmalonyl‐CoA, and ethylmalonyl‐CoA are often utilized to grow the polyketide chain. More rarely, other malonyl‐CoA derivatives can be used as substrates [6]. Given that extender units comprise a large portion of the final polyketide product, the ability to tailor ATs to incorporate non‐native extender units provides an opportunity to produce diversified polyketide products. Historically, AT engineering has been approached via domain swapping, cross‐complementation, and site‐directed mutagenesis [7, 12]. A potential alternate method, motif swapping, exchanges short sequences of amino acid residues. The substrate selectivity can be manipulated while minimally affecting the AT interactions with the rest of the PKS and the AT secondary structure. Previous mutagenesis of the well‐known YASH motif [13] (Figure 1B) has led to modest changes in extender unit promiscuity [14, 17]. Despite the YASH motif being highly conserved in methylmalonyl‐CoA type I PKSs, it is likely not completely responsible for the high native extender unit selectivity. This suggests that the remaining amino acid residues encoding extender unit selectivity have yet to be identified. Previous work in our group, guided by molecular dynamic simulations, chimeragenesis, and multisubstrate competition assays, elucidated two motifs present within EryAT6—the terminal AT in the 6‐deoxy‐erythronolide B synthase, responsible for ErA biosynthesis—that were correlated to extender unit selectivity: the large subunit motif (LSM) and the small subunit motif (SSM) (Figure 1B) [18]. To test the role of these motifs in extender unit selectivity, they were both exchanged with the corresponding motifs (Figure 1C) from the butylmalonyl‐CoA and hexylmalonyl‐CoA utilizing ATs, CinAT1 and ThaAT13 (Figure 1D) [19, 20], respectively, resulting in dramatically improved selectivity toward non‐native extender units [18].

Here, the roles of the SSM and LSM motifs were meticulously explored and dissected for the first time through mutagenesis of the EryAT6 active site. The extender unit specificity of six chimeric ATs was first probed to delineate the contribution of each motif. Then, by comparing the SSM and LSM sequences of EryAT6, CinAT1, and ThaAT13, a panel of 36 mostly novel mutants spanning 10 unique AT residues was rationally designed and tested with an established multiextender unit assay to delineate the role of specific AT residues in controlling extender unit specificity. The in vitro characterization of the extender unit specificity of the motif mutants identified several previously uncharacterized mutations associated with selectivity shifts in EryAT6. Additionally, these novel mutants were tested in pyrone‐forming single‐module extension reactions, including AT domain‐swapped Ery6 chimeras for the first time. This extensive AT mutagenesis campaign expands the known molecular determinants of extender unit specificity and paves the way for the development of PKSs to facilitate the production of tailored new‐to‐nature polyketides [21].

## 2 Results and Discussion

### 2.1 Individual Motif Swaps in AT6 of Ery6TE

**FIGURE 2:**
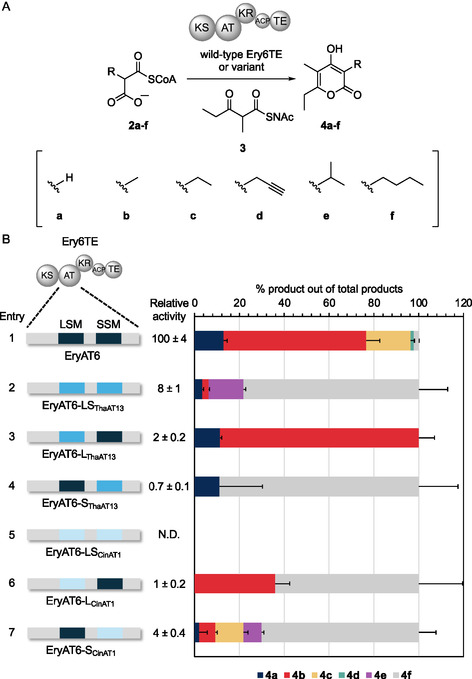
Multisubstrate competition assay of wild‐type and motif‐exchanged Ery6TE variants. (A) Scheme showing the competition assay reaction with equimolar ratios of extender units **2a–** **f** and acceptor **3**. SNAc = *N*‐acetylcysteamine. (B) Product distributions catalyzed by wild‐type and motif‐swapped Ery6TE variants determined by low‐resolution LC‐MS. Error bars are the standard deviation (*n* = 3 biological replicates) of the mean. The relative activity of wild‐type Ery6TE is set to 100. Based on the LC‐MS data, the minimum detection limit is 0.27% the activity of wild‐type Ery6TE. The design of each variant is shown where dark blue represents the corresponding EryAT6 LSM (L in the chimera's name) or SSM (S in the chimera's name), blue represents the ThaAT13 LSM/SSM, and light blue represents the CinAT1 LSM/SSM.

Previously, chimeric Ery6TE modules, in which both native AT6 LSM and SSM motifs were replaced with the corresponding motifs from CinAT1 and ThaAT13, showed a marked shift in specificity toward non‐native extender units [18]. To delineate the individual role of each motif and to begin identifying the minimal set of residues required to specify the preferred extender unit substrate, a set of single‐motif swaps was first designed and tested (Table S1). Each chimeric Ery6TE was expressed in *E. coli*, and the clarified cellular lysate (Figure S1) was incubated with **3**, a mixture of six different alkyl malonyl‐CoA extender units **2a–** **f** (Figure 2A), and the corresponding products **4a–** **f** were quantified by low‐resolution liquid chromatography with tandem mass spectrometry (LC‐MS) analysis of the crude product mixtures (Tables S2, S3 and Figure S2).

As expected [15, 18], the wild‐type EryAT6 in Ery6TE was selective toward **2b**, as judged by the distribution of pyrone products (Figure 2B; entry 1). All other extender units (except **2e**) were also incorporated, albeit at lower levels than with **2b**. As expected from previous work, swapping the LSM and SSM from ThaAT13 into EryAT6 to create EryAT6‐LS_ThaAT13_ in Ery6TE shifted **4f** production to ~80% of the total products (Figure 2B; entry 2) and supported the highest proportion of **4e** among those chimeras tested, comprising ~16% of the total products and a >10‐fold increase compared to Ery6TE. Even though it's overall activity was reduced >10‐fold compared to wild‐type EryAT6, the relative abundance of **4f** produced by EryAT6‐LS_ThaAT13_ was almost 3‐fold higher than the wild‐type enzyme (Table S3). Interestingly, substituting just the EryAT6 LSM with that from ThaAT13 resulted in less extender unit flexibility than EryAT6 (Figure 2B; entry 3), with an increased fraction of **4b**. In contrast, the corresponding ThaAT13 SSM exchange supported a significant shift in extender unit selectivity. The proportion of **4f** was >40‐fold higher than EryAT6 (Figure 2B; entry 4). The vast difference in extender unit selectivity between EryAT6‐L_ThaAT13_ and EryAT6‐S_ThaAT13_ suggested that the SSM sequence is responsible for nearly all **2f** selectivity in the double motif exchanged EryAT6‐LS_ThaAT13_ (compare entries 1–4, Figure 2B). Since ThaAT13 natively incorporates **2f** into thailandin, the SSM of ThaAT13 appears sufficient to transplant this specificity into EryAT6, albeit with a significant loss of overall activity.

Unexpectedly, introducing the CinAT1 LSM and SSM into EryAT6 resulted in an inactive chimera (Figure 2B; entry 5). In contrast, swapping in the LSM alone from CinAT1 provided a more significant shift in extender unit selectivity (Figure 2B; entry 6). Although production of **4b** remained high (~ 32%), the major product was **4f** (~57%). Swapping the EryAT6 SSM with that from CinAT1 increased the combined proportion of **4f** and **4e** by 40‐fold (Figure 2B; entry 7), accounting for ~78% of the total product. Furthermore, although the overall activity of EryAT6‐S_CinAT1_ was only ~4% that of the wild‐type enzyme, the relative abundance of **4f** was unchanged compared to that of the wild‐type enzyme. Interestingly, the calculation of the volumes and surface areas of likely catalytic pockets using CASTp and AlphaFold2‐generated protein structures as templates indicated that the EryAT6‐LS_CinAT1_ catalytic pocket was substantially larger than Ery6AT6 (Table S4). Perhaps this was not well tolerated by the overall protein structure, leading to the reduction in activity [22]. The consistent shift toward incorporating longer substituted malonyl‐CoA derivatives via SSM‐exchanged chimeras suggests that information within the SSM may be sufficient for diversifying polyketides, especially if overall activity can be improved.

### 2.2 Investigating Single Amino Acid Exchanges Within the LSM and SSM of EryAT6

Single residues in the EryAT6 LSM and SSM were exchanged with those from the CinAT1 and ThaAT13 motifs to identify residues responsible for the extender unit specificity. Comparing EryAT6 to CinAT1, 9 of the 16 amino acids throughout the LSM and SSM were not conserved (Figure 1C), while there were 6 unique amino acids compared to ThaAT13. Comparing the motifs between all three ATs (EryAT6, CinAT1, and ThaAT13), only four positions were unique amino acids: 612 (Val_EryAT6_, Gly_CinAT1_, Leu_ThaAT13_), 615 (Val_EryAT6_, Gln_CinAT1_, Asn_ThaAT13_), 739 (Thr_EryAT6_, Ala_CinAT1_, Val_ThaAT13_), and 744 (Tyr_EryAT6_, Arg_CinAT1_, Ala_ThaAT13_). To test whether these residues contribute to extender unit selectivity, the corresponding residues at positions 612, 615, and 739 in EryAT6 were targeted for mutagenesis and replaced with the corresponding residues in CinAT1 and ThaAT13 (Figure 1C and Table 1). Only the substitution to Arg was tested at position 744 (the same as previously identified Y744R). Additionally, the mutants P741R and D743E were constructed to exchange residues with those of CinAT1, given that mutating these sites to Ala shifted selectivity toward **2f** [18]. The final mutation constructed was S746G, as glycine is present at this site in CinAT1 and ThaAT13.

**TABLE 1:** Multisubstrate competition assay of wild‐type and single amino‐exchanged Ery6TE variants.

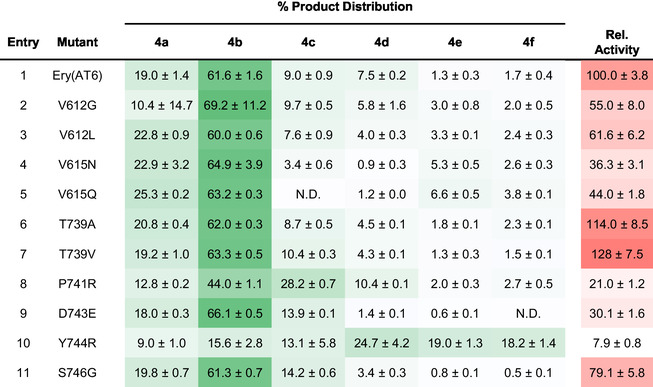

*Note:* Product distributions catalyzed by wild‐type and Ery6TE variants determined by low‐resolution LC‐MS. See Figure 2A for the competition assay reaction scheme. The relative activity (sum of extracted ion counts for all products) of wild‐type Ery6TE is set to 100. Based on the LC‐MS data, the minimum detection limit is 0.27% the activity of wild‐type Ery6TE. Errors are the standard deviation (*n* = 3 biological replicates) of the mean.

Abbreviations: N.D., not detected.

Notably, most of the single amino acid swaps resulted in similar substrate selectivity to the wild‐type EryAT6 (Table 1, compare to entry 1), as judged by the distribution of substituted pyrones **4a–** **4f** determined by LC‐MS analysis of the reaction mixtures (Tables S2 and S4). Introducing Gly at position 612 had little effect on the selectivity, but Leu doubled the fraction of **4e** compared to the WT. The swaps between residues at position 612 all maintained a nonpolar character at the position, which may explain the lack of shift in selectivity. Substitution of Val615 with Asn or Gln improved **4e** production fourfold (Table 1; entries 4 and 5). Mutations at position 739 did not result in significant changes in selectivity, perhaps because the side chain is oriented outward toward the solvent in the model (Figure 1D). The mutant P741R (Table 1; entry 8) displayed a 3‐fold increase in **4c** production and a 1.4‐fold increase in **4d**, compared to the wild‐type. This substitution may introduce electrostatic repulsion with Lys671 and Arg846 and increase the pocket size to accommodate larger extenders. The previously described promiscuity of Y744R was reproduced (Table 1; entry 10), resulting in increased selectivity for **2c–f**, including approximately a 15‐fold increase in **4e** and >10‐fold in **4f** production, compared to the wild‐type enzyme. This mutant showed the largest shift in extender unit specificity among all the single mutants tested here. The two exchanges D743E and S746G seemed to have little effect on extender unit selectivity, as judged by the product distributions (Table 1; entries 9 and 11).

### 2.3 Double and Triple Amino Acid Exchanges Within the SSM

The modest changes to extender unit selectivity of the single amino acid substitutions suggest that the large selectivity shift of the motif chimeras is likely dictated by several residues within the LSM and/or SSM. To test this, double‐ and triple‐amino acid swaps were constructed. The mutant panel was limited to the exchange of residues between the SSM of EryAT6 and CinAT1, given that (1) it would reduce the number of mutant combinations to be tested, (2) the SSM has a more prominent role in selectivity than the LSM, and (3) mutations in the SSM were relatively well tolerated (Table 1). Five residues between the EryAT6 and CinAT1 SSM were nonconserved (Figure 1C) and were targeted for mutagenesis in EryAT6. Accordingly, every possible double and triple combination of these five amino acid changes (Table 2) was constructed, providing a panel of 10 double amino acid exchanges and 10 triple amino acid mutants, which were tested using the extender unit competition assay (Figure 2A, Tables S2 and S6).

**TABLE 2:** Multisubstrate competition assay of wild‐type and multiple amino‐exchanged Ery6TE variants.

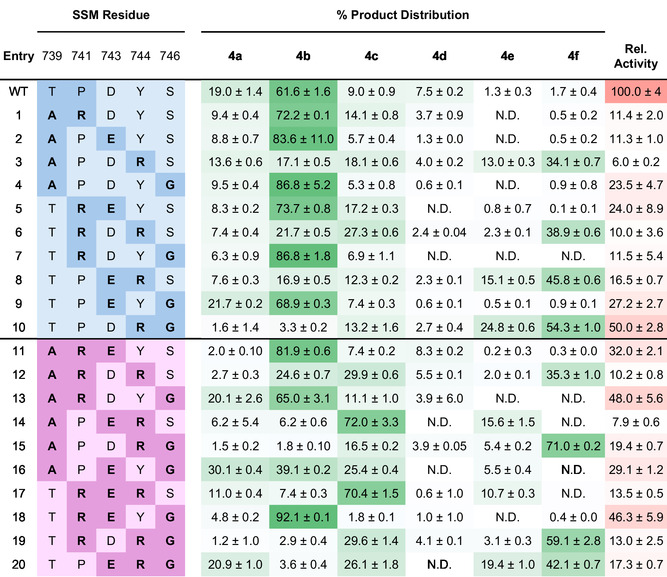

*Note:* Product distributions catalyzed by wild‐type and Ery6TE variants determined by low‐resolution LC‐MS. See Figure 2A for the competition assay reaction scheme. The relative activity (sum of extracted ion counts for all products) of wild‐type Ery6TE is set to 100. Based on the LC‐MS data, the minimum detection limit is 0.27% the activity of wild‐type Ery6TE. Errors are the standard deviation (*n* = 3 biological replicates) of the mean. N.D., not detected.

Abbreviations: N.D., not detected.

Notably, several double mutants showed distinct shifts in extender unit selectivity and promiscuity, as evidenced by product distributions. The combination of Y744R with any other single mutation tested consistently shifts selectivity toward extender units larger than **2a**/**1b** (Table 2; entries 3, 6, 8, 10, 12, 14, 15, 17, 19, and 20). However, when Y744R is paired with T739A, P741R, D743E, or S746G, the fraction of **4f** increased the most (2–3‐fold) compared to Y744R alone (Table 2; entries 3, 6, 8, and 10). The combination of P741R and Y744R maintained the **4c** production fraction observed with P741R alone, but cooperatively increased the portion of **4f** (Table 2; entry 6). Most notably, the combination of Y744R and S746G led to **4f** as the major product, suggesting that Tyr744 and Ser746 in the “YASH” motif contributed significantly to extender unit specificity. Other double mutants provided subtle shifts in selectivity. For example, P741R/D743E (Table 2; entry 5) resulted in a 2‐fold increase in the proportion of **4c** compared to WT. Other mutants displayed an increase in the fraction of **4b** compared to the wild‐type enzyme, including T739A/P741R, T739A/D743E, T739A/S746G, P741R/D743E, P741R/S746G, and D743E/S746G (Table 2; entries 1, 2, 4, 5, 7, and 9). Although improved **2f** incorporation was the highlight from the four Y744R‐containing double mutants, incorporation of the isopropyl extender unit **2e** was also enhanced by at least 10‐fold in three mutants (Y744R/ T739A, Y744R/D743E, Y744R/S746G) compared to the wild‐type enzyme (Table 2; entries 3, 8, and 10).

The triple mutants showed the most significant shifts in selectivity to date. Most notably, T739A/Y744R/S746G (Table 2; entry 15) resulted in the highest fraction of **4f** tested so far (71%), a 42‐fold and 4‐fold increase compared to the wild‐type and Y744R mutant enzyme, respectively. Moreover, T739A/Y744R/S746G improved the relative abundance of **4f** almost 2‐fold compared to the wild‐type enzyme (Table S6). Comparison to the product distributions of the contributing double mutants (T739A/Y744R and Y744R/S746G) suggests some additivity. The T739A/Y744R/S746G mutant achieved a similar proportion of the **4f** product to the EryAT6‐S_CinAT1_ chimera, but with almost fivefold higher overall activity. The triple mutant combinations also displayed changes in the fraction of **4c–** **e**. For instance, T739A/D743E/Y744R and P741R/D743E/Y744R (Table 2; entries 14 and 17) resulted in ~ 8‐fold higher production of **4c**, compared to wild‐type. They also increased **4e** production 12‐fold and 8‐fold, respectively, compared to the wild‐type. The triple mutant D743E/Y744R/S746G (Table 2; entry 20) was particularly promiscuous with one of the smallest factions of **4b** (~ 4%) and a relatively even distribution of **4c** (~ 26%), **4e** (~ 19%), and **4f** (~ 42%). Together, this data implicates several residues in the SSM in controlling extender unit specificity. Interestingly, these include residues beyond Tyr744 in the “YASH” motif, which is typically associated with specificity, and suggests that specificity is dictated by a larger number of residues, some of which reside in the SSM and LSM.

### 2.4 Exchange of Domains and Motifs Within Ery6TE

**FIGURE 3:**
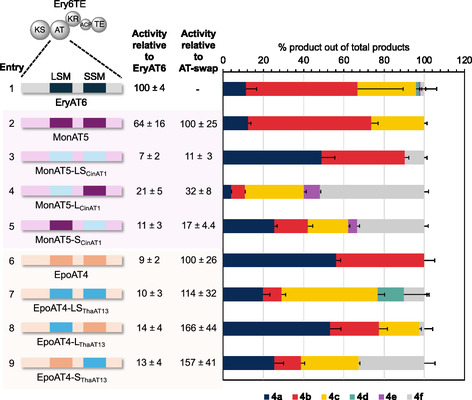
Multisubstrate competition assay of wild‐type and domain/motif‐exchanged Ery6TE variants. Product distributions catalyzed by wild‐type Ery6TE and variants determined by low‐resolution LC‐MS. Error bars are the standard deviation (*n* = 3 biological replicates) of the mean. The relative activity (sum of extracted ion counts for all products) of wild‐type Ery6TE is set to 100. Based on the LC‐MS data, the minimum detection limit is 0.27% the activity of wild‐type Ery6TE. The design of each variant is shown. Purple represents the corresponding MonAT5 LSM or SSM, orange represents the EpoAT4 LSM/SSM, light blue represents the CinAT1 LSM/SSM, and blue represents the ThaAT13 LSM/SSM (see Figure 2).

To further define the ability of the LSM and SSM to confer extender unit selectivity, the motifs from CinAT1 and ThAT13 were transplanted into Ery6TE chimeras that contained **2b**/**2c**‐specific MonAT5 [10, 23, 24] and the **2a**/**2b**‐specific EpoAT4 [14] inserted in place of the EryAT6. The goal was to determine whether the CinAT1/ThaAT13 motifs were sufficient to transfer the corresponding specificity to larger extender units in MonAT5 and EpoAT4, leveraging the Ery6TE chimera for convenient assays of pyrone formation. Interestingly, consistent with the implication of SSM residues from previous site‐directed mutagenesis of EryAT6, SSM residues are poorly conserved among EryAT6, MonAT5, and EpoAT4 (Figure 1C). To establish a baseline for selectivity, the MonAT5 and EpoAT4 domain swaps were first tested (entries 2 and 6, Figure 3, Tables S2 and S7). As expected, based on the known specificity of MonAT5, swapping AT6 with MonAT5 in Ery6TE produced **4b** (61%) and **4c** (26%) as the only products (compare entries 1 and 2, Figure 3), while **4d–** **4f** were not detected. Similarly, the EpoAT4 domain swap only produced **4a** (56%) and **4b** (44%) (Figure 3; entries 2 and 6), with no detection of **4c–** **4f**. Notably, although the product distributions from these domain swaps validated their native selectivity, overall activity was diminished, as expected due to disrupted protein folding and/or protein interactions within the AT‐swapped chimera. Next, the LSM and SSM of each chimeric Ery6TE module were swapped with those from CinAT1 or ThaAT13, furnishing 12 constructs in total. However, 5 of the 12 constructs could not be expressed, likely due to incompatibilities associated with the AT domain swaps. In the MonAT5 swap, every combination of CinAT1 motifs was tested. Compared to Ery6(MonAT5)TE (Figure 3; entry 2), the double motif‐swapped Ery6(MonAT5‐LS_CinAT1_)TE, product profile shifted toward **4a** (Figure 3; entry 3). The increased proportion of **4a** was unexpected but followed a similar trend as Ery6(AT6‐LS_CinAT1_)TE, where the only product observed was **4b** (Figure 2; entry 5). The single motif swapped Ery6(MonAT5‐L_CinAT1_)TE displayed a significant portion of **4f** (52%) and **4e** (Figure 3; entry 4). It showed a similar proportion of **4c** compared to Ery6(MonAT5)TE. Given that the MonAT5 swap did not produce **4f**, exchanging the LSM with the CinAT1 LSM increased relative abundance of **4f** at least 20‐fold (Table S7), representing an inversion of extender unit specificity. In contrast, the analogous chimera using the SSM of CinAT1 led to modest increases in the portions of **4a**, **4e**, and **4f** (Figure 3; entry 5). Thus, the LSM and SSM from CinAT1 affect extender unit selectivity when individually installed into the MonAT5/Ery6TE chimera, enabling the production of **4f** and inverting the substrate specificity. Interestingly, substitution with the CinAT1 SSM led to a more promiscuous chimera, whereas substitution with the CinAT1 LSM led to a more **4f**‐specific chimera, as judged by the product distributions. Given that the reference MonAT5 swap does not produce detectable **4f**, each of the MonAT5 motif‐swapped chimeras (Figure 3 and Table S7; entries 3–5) produced a higher relative abundance of **4f** than the reference MonAT5 swap (entry 2), and most of them produced more **4f** compared to the wild‐type EryAT6, even with reduced overall activities.

Three Ery6(EpoAT4)TE motif‐swapped chimeras were tested. In contrast to Ery6(EpoAT4)TE, which did not support production of **4c**, 48% of the pyrone produced by the double motif‐swapped Ery6(EpoAT4‐LS_ThaAT13_)TE was the ethyl product (Figure 3; entry 7), with an increase in the proportion of **4d** and **4f** also observed. Similarly, both single motif‐swapped chimeras, Ery6(EpoAT4L_ThaAT13_)TE and Ery6(EpoAT4‐S_ThaAT13_)TE, enabled **4c** and **4f** production (Figure 3, entries 8 and 9). Notably, with Ery6(EpoAT4‐S_ThaAT13_), the most abundant product was **4f** (~ 32%), closely followed by **4a** (~25%) and **4c** (~29%) (Figure 3, entries 8 and 9), representing a significant shift in specificity from the parent AT‐swap (Figure 3, compare entries 6 and 9). Notably, the overall activity of every motif swap in the Ery6(EpoAT4)TE was indistinguishable from the AT‐swapped parent, suggesting that the motif‐swapping approach has the potential to manipulate extender unit specificity while maintaining robust activity. Similar to the MonAT5 series, given that **4f** is not detectable with the reference EpoAT5 swap, each of the EpoAT5 motif‐swapped chimeras (Figure 3 and Table S7; entries 7–9) produced a higher relative abundance of **4f** than the reference EpoAT5 swap (entry 6) and wild‐type EryAT6 (entry 1).

In the absence of domain‐swapped PKS module structures, the volumes and surface areas of likely catalytic pockets were calculated using CASTp and AlphaFold2‐generated protein structures as templates (Table S4) [22]. The predicted catalytic pocket volume of the EryAT6(MonAT5) chimera was similar to that of EryAT6 (Table S4), consistent with the similar product distributions catalyzed by these modules (Figure 3). Furthermore, the EryAT6(MonAT5) motif‐swap with the largest shift toward **4f** (Entry 4, Figure 3) also displayed the largest calculated catalytic pocket volume. In general, the catalytic pocket volume of the swaps involving EpoAT4 was smaller than that of MonAT5, consistent with the native extender unit specificity of EpoAT4 versus MonAT5. Yet, the pocket volume of the EpoAT4 chimera with the ThaAT13 SSM was the largest, and this chimera also displayed the largest shift toward **4f** (Entry 9, Figure 3). Therefore, consistent with a previous study of AT‐swapped Ery6 chimeras [10], the active‐site pocket volume appears to correlate with the chimeric Ery6 module's ability to utilize extender units with larger side chains. Testing the SSM and LSM in the context of AT‐swapped Ery6TE chimeras demonstrated that the LSM and SSM motifs can dramatically shift extender selectivity in ATs from monensin and epothilone biosynthesis. The data also suggest that the SSM confers greater promiscuity on the target AT than the LSM.

## 3 Conclusions

Leveraging PKSs to accept non‐native extender units has been explored using various strategies [25, 31], including AT‐domain swapping with newly defined boundaries [10]. Yet, aside from their role in predicting extender unit specificity of PKS AT domains, little is known about the sequence determinants underlying extender unit selectivity and how they can be manipulated. This has largely restricted AT engineering efforts to replacing the entire AT, which, even with updated domain boundaries, does not always lead to robust activities [10, 32], presumably due to the disruption of critical protein structure, protein–protein interactions, conformational dynamics, and substrate channeling.

Herein, we dissected the contributions of the recently described motifs SSM and LSM to extender unit selectivity and used this information to invert the product distributions of mono‐modular PKSs. First, we discovered that in Ery6TE, exchanging the SSM with those from CinAT1 and ThaAT13, which both prefer longer chain extender units such as **2f**, was sufficient to shift extender unit selectivity toward larger extender units. This suggests that the SSM contains enough sequence information to dictate selectivity. This was underscored by site‐directed mutagenesis, which demonstrated that triple amino acid exchanges between the SSM in Ery6TE and the corresponding residues in CinAT1 yielded the largest shifts in extender unit selectivity and largely recapitulated the product distributions catalyzed by the single motif‐swapped chimera. This suggests that extender unit selectivity could be determined by a relatively small number of residues in the SSM. Overall, this revealed a minimum set of residues in EryAT6 that potentially dictate extender unit selectivity and, when mutated to their CinAT1 counterparts, can shift selectivity. These EryAT6 residues include the newly identified T739A, P741R, D743E, and S746G, as well as the previously identified Y744R. Cooperativity between mutations is essential, given that the largest shifts in extender unit selectivity were observed with some of the triple mutants.

The role of the SSM in dictating extender unit selectivity was further demonstrated by exchanging the SSM in AT‐swapped Ery6TE chimeras with the SSM from CinAT1 and ThaAT13. Notably, these AT‐swapped chimeras were used here as convenient benchmarks, leveraging their ability to access substituted pyrones, which can be easily detected. Remarkably, exchanging the CinAT1 and ThaAT13 SSMs into the AT‐swapped chimeras resulted in the production of butyl‐substituted pyrones, even though the AT‐swaps alone could not support their production, representing, for example, at least a 20‐fold increase in relative abundance over the MonAT5 swap. Considering these variants comprise exchanges of the AT domains and SSMs, their activities relative to wild‐type Ery6TE were quite good. We speculate that exchanging the SSM directly within a target PKS module will likely yield improved relative activity.

These studies advance our understanding of AT extender unit selectivity and lay the foundation for reprograming PKSs with customized extender unit specificity. The exchange of SSMs between ATs with unique extender unit specificities and/or the targeted exchange of amino acid residues between them presents a strategy for expanding chemical diversity in polyketide biosynthesis that complements other approaches. Future studies will focus on integrating motif‐swapped ATs into other PKS systems, including full‐length PKSs, to further assess and validate their applicability for producing a diverse range of polyketide scaffolds.

## 4 Materials and Methods

### 4.1 General Information

Materials and reagents were purchased from Sigma Aldrich unless otherwise noted. Isopropyl β‐D‐thiogalactoside (IPTG) was purchased from Calbiochem. All holo proteins were expressed in *E. coli* K207‐3 cells containing sfp (Table S8), provided by Dr. Adrian Keatinge‐Clay at the University of Texas at Austin. Primers were purchased from Integrated DNA Technologies. All oligo sequences are listed in Table S9.

### 4.2 Chemoenzymatic Preparation of Acyl‐CoA Extender Units

Wild‐type MatB and the mutant MatB or T207G/M306I were purified (See Supplemental Methods), and ~8 mM stocks of the malonyl‐CoA analogs were prepared as previously described [15, 18, 33].

### 4.3 Ery6TE Lysate Preparation

Wild‐type and mutant Ery6TE mutant plasmids in *E. coli* K207‐3 were expressed overnight in 300 mL of LB broth containing 30 μL/mL Kan. Protein production was induced when the culture reached an OD_600_ of ~0.6 with the addition of IPTG to 1 mM and was then incubated at 18°C for 20 h with shaking at 250 rpm. Cells were then collected by centrifugation at 4,800 *g* at 4°C for 15 min. The supernatant was discarded, and the resulting cell pellet was resuspended in a module storage buffer containing 100 mM Sodium Phosphate and 1 mM EDTA. Resuspended cells were lysed by sonication, and the cell lysate was clarified by centrifugation at 10,000 *g* and 4°C for 1 h. Cell lysates containing the Ery6TE protein were stored at 10% glycerol at −80°C. Expression of the target protein was verified by sodium dodecyl sulfate‐polyacrylamide gel electrophoresis (SDS‐PAGE). Protein quantification was carried out using the Bradford Protein Assay Kit from Bio‐Rad.

### 4.4 Ery6TE Extender Unit Competition Assay

The Ery6TE pyrone assays were set up in a total volume of 50 μL of 100 mM sodium phosphate, pH 7.2, 20% (v/v) glycerol, 0.8% DMSO, and 2 mM dithiothreitol. The reaction conditions included 10 mM diketide‐SNAC (**3**), 1 mM each extender unit (**2a–** **2f**), and 1 mM Ery6TE lysate. Module concentrations were determined by Bradford assay and SDS‐PAGE, and results were normalized to protein content for each reaction. Reactions were performed at 22°C for 24 h, quenched with an equal volume of ice‐cold methanol, and stored at −80°C overnight. Reactions were then centrifuged at 10,000 *g* for 30 min at 4°C. The supernatant containing the pyrone was removed and centrifuged under the same conditions for an additional 30 min. Clarified samples were loaded into 1.5 mL glass vials containing 200 μL vial inserts for LC‐MS analysis. Analysis was carried out on a low‐resolution Shimadzu LCMS‐2050 equipped with a PDA (SPD‐M40). The mobile A phase was LC‐MS grade water supplemented with 0.1% formic acid (Thermo Scientific). The mobile B phase was LC‐MS grade acetonitrile. The LC column was a Shimadzu Shim‐pack XR‐ODS 3.0 × 100 mm, 2.2 μm particle size. Samples were analyzed using an 8‐min method, comprising a linear gradient of 5%–95% B (0–6.1 min), 95% B (6.1–6.6 min), 5% B (6.6–8 min). The mass spectrometer was operated in positive ion mode. The assay produces pyrone products that can be detected as their [M+H]^+^ ions. Peak areas for extracted ions were used to calculate product peak areas. Every assay was repeated three distinct times, unless otherwise stated. For retention times, calculated masses, observed masses, representative extracted ion counts, and representative chromatograms, see Tables S2–S6, and Figure S2. In every case, the retention times were consistent with previous examples [15, 18, 34, 35] and were compared with boiled enzyme controls.

## Author Contributions

S.W. and G.J.W. conceived the project. S.W. designed and executed all Ery6 engineering and assays. All authors analyzed the data. G.J.W. supervised the research. All authors wrote the manuscript.

## Funding

This study was supported by the National Institute of General Medical Sciences (GM104258).

## Conflicts of Interest

The authors declare no conflicts of interest.

## Supporting information

Supplementary Material

## Data Availability

The data that support the findings of this study are available in the Supporting Information of this article. Additional raw data are available from the corresponding author upon reasonable request.
