# Supplementary material for: Dissection and Engineering of Modular Polyketide Synthase Extender Unit Specificity Motifs
Source: Chembiochem. 2026 Jul 10;27(13):e70447. doi: 10.1002/cbic.70447 (PMC13351777; doi:10.1002/cbic.70447)
Supplement: Supplementary file 1 — Supplementary Material [file CBIC-27-e70447-s001.pdf]

Dissection and engineering of modular polyketide synthase extender unit specificity motifs

Sydney Welch[1,2] and Gavin J Williams[1,3]*

[1] Department of Chemistry, NC State University, Raleigh, NC 27695-8204, United States

[2] Present address: KBI BioPharma, 4117 Emperor Blvd, Durham, North Carolina, 27703, United States

[3] Comparative Medicine Institute, NC State University, Raleigh, NC, United States

* Corresponding author, gjwillia@ncsu.edu

TABLE OF CONTENTS

__Supplemental Tables__

Supplementary Table S1. Nucleotide sequence for wild-type Ery6TE and motif/domain-swapped chimeras.

Supplementary Table S2. Low-resolution LC-MS retention times, calculated masses, and observed masses for Ery6TE-catalyzed reaction products.

Supplementary Table S3. Low-resolution peak areas for Ery6TE reaction products with competing extender units.

Supplementary Table S4. CASTp 3.0 active site surface area and volume calculations of EryAT6 domain/motif-swapped variants.

Supplementary Table S5. Low-resolution peak areas for Ery6TE single mutant enzyme reaction products with competing extender units.

Supplementary Table S6. Low-resolution peak areas of Ery6TE double and triple mutant enzyme-catalyzed reaction products with competing extender units.

Supplementary Table S7. Low-resolution peak areas of AT domain/motif-exchanged Ery6TE variant-catalyzed reaction products with competing extender units.

Supplementary Table S8. *E. coli* strains used in this study.

Supplementary Table S9. Oligonucleotides used in this study.

__Supplemental Figures__

Supplementary Figure S1. SDS-Page analysis of clarified cellular lysates of expressed Ery6TE modules.

Supplementary Figure S2. Representative extracted ion chromatograms of Ery6TE-catalyzed reactions.

__Supplemental Methods__

Expression and Purification of Wild-Type and Mutant MatB Enzymes.

Construction of Ery6TE Single/Double/Triple Mutants.

Construction of Ery6TE Single Motif Swaps.

Construction of Ery6TE AT Swaps.

Modeling of EryAT6 Motif Swaps and CASTp Analysis.

<u>**Supplemental Tables**</u>

Supplementary Table S1. Nucleotide sequence for wild-type Ery6TE and motif/domain-swapped chimeras.

Gene	Sequence
**Wild-type Ery6TE**<br>Highlighted in red is the AT6 sequence.<br>Highlighted in **green** is the large subunit motif and in **blue** is the small subunit motif.	ATGACGAGTTCCAACGAACAGTTGGTGGACGCTCTGCGCGCCTCTCTCAAGGAGAAC<br>GAAGAACTCCGGAAAGAGAGCCGTCGCCGGGCCGACCGTCGGCAGGAGGAGATCGCG<br>ATCGTCGGCATGGCCTGCCGCTTCCCCGGCGGCGTGCACAACCCCGGTGAGCTGTGG<br>GAGTTCATCGTCGGCGGCGGAGACGCCGTGACGGAGATGCCCACCGACCGCGGCTGG<br>GACCTCGACGCGCTGTTCGACCCCGACCCGCAGCGCCACGGAACCAGCTACTCGCGA<br>CACGGCGCGTTCCTCGACGGGGCCGCCGACTTCGACGCGGCGTTCTTCGGGATCTCG<br>CCGCGCGAGGCGCTGGCGATGGACCCGCAGCAGCGCCAGGTCCTGGAAACGACGTGG<br>GAGCTGTTCGAGAACGCCGGCATCGACCCGCACTCGCTGCGGGGCAGCGACACCGGC<br>GTCTTCCTCGGCGCCGCGTACCAGGGCTACGGCCAGGACGCGGTGGTGCCCGAGGAC<br>AGCGAGGGCTACCTGCTCACCGGCAACTCCTCCGCCGTGGTGTCCGGCCGGGTCGCC<br>TACGTGCTGGGGCTGGAAGGCCCCGCGGTCACGGTGGACACGGCGTGTTCGTCGTCG<br>TTGGTGGCCTTGCATTCGGCGTGTGGGTCGTTGCGTGACGGTGACTGCGGTCTTGCG<br>GTGGCCGGTGGTGTGTCGGTGATGGCGGGCCCGGAGGTGTTCACCGAGTTCTCCCGC<br>CAGGGCGGCTTGGCCGTGGACGGGCGCTGCAAGGCGTTCTCCGCGGAGGCCGACGGC<br>TTCGGTTTCGCCGAGGGCGTCGCGGTGGTCCTGCTCCAGCGGTTGTCCGACGCCCGC<br>AGGGCGGGTCGCCAGGTGCTCGGCGTGGTCGCGGGCTCGGCGATCAACCAGGACGGC<br>GCGAGCAACGGTCTCGCGGCGCCGAGCGGCGTCGCCCAGCAGCGCGTGATCCGCAAG<br>GCGTGGGCGCGTGCGGGGATCACGGGCGCGGATGTGGCCGTGGTGGAGGCGCATGGG<br>ACCGGTACGCGGCTGGGCGATCCGGTGGAGGCGTCGGCGTTGCTGGCTACTTACGGC<br>AAGTCGCGCGGGTCGTCGGGCCCGGTGCTGCTGGGTTCGGTGAAGTCGAACATCGGT<br>CACGCGCAGGCGGCCGCGGGTGTCGCGGGCGTGATCAAGGTGGTCCTGGGGTTGAAC<br>CGCGGCCTGGTGCCGCCGATGCTCTGCCGCGGCGAGCGGTCGCCGCTGATCGAATGG<br>TCCTCGGGTGGTGTGGAACTTGCCGAGGCCGTGAGCCCGTGGCCTCCGGCCGCGGAC<br>GGGGTGCGCCGGGCCGGTGTGTCGGCGTTCGGGGTGAGCGGGACGAACGCGCACGTG<br>ATCATCGCCGAGCCCCCGGAGCCCGAGCCGCTGCCGGAACCCGGACCGGTGGGCGTG<br>CTGGCCGCTGCGAACTCGGTGCCCGTACTGCTGTCGGCCAGGACCGAGACCGCGTTG<br>GCAGCGCAGGCGCGGCTCCTGGAGTCCGCAGTGGACGACTCGGTTCCGTTGACGGCA<br>TTGGCTTCCGCGCTGGCCACCGGACGCGCCCACCTGCCGCGTCGTGCGGCGTTGCTG<br>GCAGGCGACCACGAACAGCTCCGCGGGCAGTTGCGAGCGGTCGCCGAGGGCGTTGCG

	GCTCCCGGTGCCACCACCGGAACCGCCTCCGCCGGCGGCGTGGTTTTCGTCTTCCCA<br>GGTCAGGGTGCTCAGTGGGAGGGCATGGCCCGGGGCTTGCTCTCGGTCCCCGTCTTC<br>GCCGAGTCGATCGCCGAGTGCGATGCGGTGTTGTCGGAGGTGGCCGGGTTCTCGGCC<br>TCCGAAGTGCTGGAGCAGCGTCCGGACGCGCCGTCGCTGGAG**CGGGTCGACGTCGTA**<br>**CAGCCG**GTGTTGTTCTCCGTGATGGTGTCGCTGGCGCGGCTGTGGGGCGCTTGCGGA<br>GTCAGCCCCTCGGCCGTCATCGGCCATTCGCAGGGCGAGATCGCCGCCGCGGTGGTG<br>GCCGGGGTGTTGTCGCTGGAGGACGGCGTGCGCGTCGTGGCCCTGCGCGCGAAGGCG<br>TTGCGTGCGCTGGCGGGCAAGGGCGGCATGGTCTCGTTGGCGGCTCCCGGTGAACGC<br>GCCCGCGCGCTGATCGCACCGTGGGAGGACCGGATCTCCGTCGCGGCGGTCAACTCC<br>CCGTCCTCGGTCGTGGTCTCCGGCGATCCGGAGGCGCTGGCCGAACTCGTCGCACGT<br>TGCGAGGACGAGGGCGTGCGCGCCAAG**ACGCTCCCGGTGGACTACGCCTCGCAC**TCC<br>CGCCACGTCGAGGAGATCCGCGAGACGATCCTCGCCGACCTCGACGGCATCTCCGCG<br>CGGCGTGCCGCCATCCCGCTCTACTCCACGCTGCACGGCGAACGGCGCGACGGCGCC<br>GACATGGGTCCGCGGTACTGGTACGACAACCTGCGCTCCCAGGTGCGCTTCGACGAG<br>GCGGTCTCGGCCGCCGTCGCCGACGGTCACGCCACCTTCGTCGAGATGAGCCCGCAC<br>CCGGTGCTCACCGCGGCGGTGCAGGAGATCGCCGCGGACGCCGTGGCCATCGGGTCG<br>CTGCACCGCGACACCGCGGAGGAGCACCTGATCGCCGAGCTCGCCCGGGCGCACGTG<br>CACGGCGTGGCCGTGGACTGGCGGAACGTCTTCCCGGCGGCACCTCCCGGTGGCGTG<br>CCCAACTACCCGTTCGAGCCCCAGCGGTACTGGCTCGCGCCGGAGGTGTCCGACCAG<br>CTCGCCGACAGCCGCTACCGCGTCGACTGGCGACCGCTGGCCACCACGCCGGTGGAC<br>CTGGAAGGCGGCTTCCTGGTCCACGGGTCCGCACCGGAGTCGCTGACCAGCGCAGTC<br>GAGAAGGCCGGAGGCCGCGTCGTGCCGGTCGCCTCGGCCGACCGCGAAGCGCTCGCG<br>GCGGCCCTGCGGGAGGTGCCGGGCGAGGTCGCCGGCGTGCTCTCGGTCCACACCGGC<br>GCCGCAACGCACCTCGCCCTGCACCAGTCGCTGGGTGAGGCCGGCGTGCGGGCCCCG<br>CTCTGGCTGGTCACCAGCCGAGCGGTCGCGCTCGGGGAGTCCGAGCCGGTCGATCCC<br>GAGCAGGCGATGGTGTGGGGTCTCGGGCGCGTCATGGGCCTGGAGACCCCGGAACGG<br>TGGGGCGGTCTGGTGGACCTGCCCGCCGAACCCGCGCCGGGGGACGGCGAGGCGTTC<br>GTCGCCTGCCTCGGCGCGGACGGCCACGAGGACCAGGTCGCGATCCGTGACCACGCC<br>CGCTACGGCCGCCGCCTCGTCCGCGCCCCGCTGGGCACCCGCGAGTCGAGCTGGGAG<br>CCGGCGGGCACGGCGCTGGTCACCGGCGGCACCGGTGCGCTCGGCGGCCACGTCGCC<br>CGCCACCTCGCCAGGTGCGGGGTGGAGGACCTGGTGCTGGTCAGCAGGCGCGGCGTC<br>GACGCTCCCGGCGCGGCCGAGCTGGAAGCCGAACTGGTCGCCCTCGGCGCGAAGACG<br>ACCATCACCGCCTGCGACGTGGCCGACCGCGAGCAGCTCTCCAAGCTGCTGGAAGAA<br>CTGCGCGGGCAGGGACGTCCGGTGCGGACCGTCGTGCACACCGCCGGGGTGCCCGAA

	TCGAGGCCGCTGCACGAGATCGGCGAGCTGGAGTCGGTCTGCGCGGCGAAGGTGACC<br>GGGGCCCGGCTGCTCGACGAGCTGTGCCCGGACGCCGAGACCTTCGTCCTGTTCTCG<br>TCCGGAGCGGGGGTGTGGGGCAGTGCGAACCTCGGCGCCTACTCCGCGGCCAACGCC<br>TACCTCGACGCGCTGGCCCACCGCCGCCGTGCGGAAGGCCGTGCGGCGACGTCCGTC<br>GCGTGGGGCGCCTGGGCGGGCGAGGGCATGGCCACCGGCGACCTCGAGGGGCTCACC<br>CGGCGCGGCCTGCGCCCGATGGCGCCCGAGCGCGCGATCCGCGCGCTGCACCAGGCG<br>CTGGACAACGGCGACACGTGCGTTTCGATCGCCGACGTCGACTGGGAGCGCTTCGCG<br>GTCGGCTTCACCGCCGCCCGGCCGCGTCCGCTGCTGGACGAGCTCGTCACGCCGGCG<br>GTGGGGGCCGTCCCCGCGGTGCAGGCGGCCCCGGCGCGGGAGATGACGTCGCAGGAG<br>TTGCTGGAGTTCACGCACTCGCACGTCGCGGCGATCCTCGGGCATTCCAGCCCGGAC<br>GCGGTCGGGCAGGACCAGCCGTTCACCGAGCTCGGCTTCGACTCGCTGACCGCGGTC<br>GGGCTGCGCAACCAGCTCCAGCAGGCCACCGGGCTCGCGCTGCCCGCGACCCTGGTG<br>TTCGAGCACCCCACGGTCCGCAGGTTGGCCGACCACATAGGACAGCAGCTCGACAGC<br>GGGACTCCCGCCCGGGAAGCGAGCAGCGCTCTTCGCGACGGCTACCGGCAGGCGGGC<br>GTGTCGGGCAGGGTCCGGTCCTACCTCGACCTGCTGGCGGGGCTGTCGGACTTCCGC<br>GAGCACTTCGACGGCTCCGACGGGTTCTCCCTCGATCTCGTGGACATGGCCGACGGT<br>CCCGGAGAGGTCACGGTGATCTGCTGCGCGGGAACGGCGGCGATCTCCGGTCCGCAC<br>GAGTTCACCCGGCTCGCCGGGGCGCTGCGCGGAATCGCTCCGGTTCGGGCCGTGCCC<br>CAGCCCGGCTACGAGGAGGGCGAACCTCTGCCGTCGTCGATGGCGGCGGTGGCGGCG<br>GTGCAGGCCGATGCGGTCATCAGGACACAGGGGGACAAGCCGTTCGTGGTGGCCGGT<br>CACTCCGCGGGGGCACTGATGGCCTACGCGCTGGCGACCGAACTGCTCGATCGCGGG<br>CACCCGCCACGCGGTGTCGTCCTGATCGACGTCTACCCGCCCGGTCACCAGGACGCG<br>ATGAACGCCTGGCTGGAGGAGCTGACCGCCACGCTGTTCGACCGCGAGACGGTGCGG<br>ATGGACGACACCAGGCTCACCGCCCTGGGCGCCTACGACCGCCTCACCGGTCAGTGG<br>CGACCCCGGGAAACCGGGCTGCCGACGCTGCTGGTCAGCGCCGGCGAGCCGATGGGT<br>CCGTGGCCCGACGACAGCTGGAAGCCGACGTGGCCCTTCGAGCACGACACCGTCGCC<br>GTCCCCGGCGACCACTTCACGATGGTGCAGGAACACGCCGACGCGATCGCGCGGCAC<br>ATCGACGCCTGGCTGGGCGGAGGGAATTCAAGA
**MonAT5**	ATGACGAGTTCCAACGAACAGTTGGTGGACGCTCTGCGCGCCTCTCTCAAGGAGAAC<br>GAAGAACTCCGGAAAGAGAGCCGTCGCCGGGCCGACCGTCGGCAGGAGGAGATCGCG<br>ATCGTCGGCATGGCCTGCCGCTTCCCCGGCGGCGTGCACAACCCCGGTGAGCTGTGG<br>GAGTTCATCGTCGGCGGCGGAGACGCCGTGACGGAGATGCCCACCGACCGCGGCTGG<br>GACCTCGACGCGCTGTTCGACCCCGACCCGCAGCGCCACGGAACCAGCTACTCGCGA<br>CACGGCGCGTTCCTCGACGGGGCCGCCGACTTCGACGCGGCGTTCTTCGGGATCTCG

	CCGCGCGAGGCGCTGGCGATGGACCCGCAGCAGCGCCAGGTCCTGGAAACGACGTGG<br>GAGCTGTTCGAGAACGCCGGCATCGACCCGCACTCGCTGCGGGGCAGCGACACCGGC<br>GTCTTCCTCGGCGCCGCGTACCAGGGCTACGGCCAGGACGCGGTGGTGCCCGAGGAC<br>AGCGAGGGCTACCTGCTCACCGGCAACTCCTCCGCCGTGGTGTCCGGCCGGGTCGCC<br>TACGTGCTGGGGCTGGAAGGCCCCGCGGTCACGGTGGACACGGCGTGTTCGTCGTCG<br>TTGGTGGCCTTGCATTCGGCGTGTGGGTCGTTGCGTGACGGTGACTGCGGTCTTGCG<br>GTGGCCGGTGGTGTGTCGGTGATGGCGGGCCCGGAGGTGTTCACCGAGTTCTCCCGC<br>CAGGGCGGCTTGGCCGTGGACGGGCGCTGCAAGGCGTTCTCCGCGGAGGCCGACGGC<br>TTCGGTTTCGCCGAGGGCGTCGCGGTGGTCCTGCTCCAGCGGTTGTCCGACGCCCGC<br>AGGGCGGGTCGCCAGGTGCTCGGCGTGGTCGCGGGCTCGGCGATCAACCAGGACGGC<br>GCGAGCAACGGTCTCGCGGCGCCGAGCGGCGTCGCCCAGCAGCGCGTGATCCGCAAG<br>GCGTGGGCGCGTGCGGGGATCACGGGCGCGGATGTGGCCGTGGTGGAGGCGCATGGG<br>ACCGGTACGCGGCTGGGCGATCCGGTGGAGGCGTCGGCGTTGCTGGCTACTTACGGC<br>AAGTCGCGCGGGTCGTCGGGCCCGGTGCTGCTGGGTTCGGTGAAGTCGAACATCGGT<br>CACGCGCAGGCGGCCGCGGGTGTCGCGGGCGTGATCAAGGTGGTCCTGGGGTTGAAC<br>CGCGGCCTGGTGCCGCCGATGCTCTGCCGCGGCGAGCGGTCGCCGCTGATCGAATGG<br>TCCTCGGGTGGTGTGGAACTTGCCGAGGCCGTGAGCCCGTGGCCTCCGGCCGCGGAC<br>GGGGTGCGCCGGGCCGGTGTGTCGGCGTTCGGGGTGAGCGGTACGAATGCACACGTC<br>GTTTTGGAAGAGGCTCCAGCCGTTGAGTTATGGCCAGCAGCACCCGAGAGATCGGCA<br>GAGTTACTTGTACTTTCGGGTAAGTCAGAGGGAGCCTTAGACGCACAAGCTGCTCGG<br>CTTCGAGAGCACTTGGACATGCACCCAGAGTTGGGATTAGGGGACGTCGCATTCTCT<br>TTAGCCACGACACGTTCCGCAATGAATCACCGGCTTGCTGTTGCTGTAACTAGTCGT<br>GAGGGTTTACTCGCAGCACTCAGTGCCGTCGCACAAGGTCAAACACCACCTGGGGCT<br>GCACGTTGTATCGCTTCGAGTTCCCGGGGTAAGCTCGCATTCTTATTCACAGGGCAA<br>GGAGCCCAAACTCCCGGTATGGGGCGTGGTTTATGTGCTGCATGGCCCGCATTCCGT<br>GAGGCCTTCGACCGGTGTGTAGCTCTTTTCGACCGTGAGTTAGACCGTCCCTTATGT<br>GAGGTAATGTGGGCAGAGCCTGGGAGTGCAGAGTCATTGCTTCTCGACCAAACGGCC<br>TTCACGCAACCTGCCTTGTTCACTGTAGAGTACGCTCTTACAGCACTTTGGCGTTCG<br>TGGGGAGTTGAGCCCGAGTTAGTTGCCGGTCACAGTGCTGGTGAGCTTGTTGCAGCT<br>TGTGTCGCCGGTGTTTTCTCGCTTGAGGACGGTGTACGTCTCGTTGCAGCCCGGGGT<br>CGATTAATGCAAGGATTATCCGCAGGCGGGGCTATGGTTTCCCTTGGGGCACCTGAG<br>GCAGAGGTCGCCGCCGCAGTTGCACCACACGCAGCCTGGGTATCTATAGCAGCCGTT<br>AATGGACCTGAGCAAGTTGTAATAGCTGGAGTCGAGCAAGCCGTTCAAGCAATAGCA<br>GCTGGGTTCGCTGCAAGAGGTGTAAGAACGAAGCGTCTTCACGTCTCCCACGCTTCT

	CACTCTCCATTGATGGAGCCTATGTTAGAGGAGTTCGGGAGAGTTGCAGCCAGTGTT<br>ACATACCGTCGTCCTTCAGTCAGTTTAGTTTCTAATTTATCCGGTAAGGTAGTAACG<br>GACGAGCTTTCCGCACCCGGTTACTGGGTTAGACACGTCCGTGAGGCAGTACGGTTC<br>GCCGACGGTGTCAAGGCACTCCACGAGGCCGGTGCAGGTACTTTCTTAGAGGTCGGA<br>CCAAAGCCAACACTTTTGGGTTTATTACCAGCATGTTTGCCTGAGGCTGAGCCAACG<br>TTACTCGCCTCTCTTCGGGCTGGTCGGGAAGAGGCCGCCGGTGTTTTAGAGGCCTTA<br>GGTCGGTTATGGGCAGCCGGCGGTTCAGTCTCTTGGCCTGGAGTTTTCCCAACTGCA<br>GGACGACGTGTTGACCGCTGGCGGGCAAGGGCGGCATGGTCTCGTTGGCGGCTCCCG<br>GTGAACGCGCCCGCGCGCTGATCGCACCGTGGGAGGACCGGATCTCCGTCGCGGCGG<br>TCAACTCCCCGTCCTCGGTCGTGGTCTCCGGCGATCCGGAGGCGCTGGCCGAACTCG<br>TCGCACGTTGCGAGGACGAGGGCGTGCGCGCCAAG**ACGCTCCCGGTGGACTACGCCT**<br>**CGCAC**TCCCGCCACGTCGAGGAGATCCGCGAGACGATCCTCGCCGACCTCGACGGCA<br>TCTCCGCGCGGCGTGCCGCCATCCCGCTCTACTCCACGCTGCACGGCGAACGGCGCG<br>ACGGCGCCGACATGGGTCCGCGGTACTGGTACGACAACCTGCGCTCCCAGGTGCGCT<br>TCGACGAGGCGGTCTCGGCCGCCGTCGCCGACGGTCACGCCACCTTCGTCGAGATGA<br>GCCCGCACCCGGTGCTCACCGCGGCGGTGCAGGAGATCGCCGCGGACGCCGTGGCCA<br>TCGGGTCGCTGCACCGCGACACCGCGGAGGAGCACCTGATCGCCGAGCTCGCCCGGG<br>CGCACGTGCACGGCGTGGCCGTGGACTGGCGGAACGTCTTCCCGGCGGCACCTCCCG<br>GTGGCGTGCCCAACTACCCGTTCGAGCCCCAGCGGTACTGGCTCGCGCCGGAGGTGT<br>CCGACCAGCTCGCCGACAGCCGCTACCGCGTCGACTGGCGACCGCTGGCCACCACGC<br>CGGTGGACCTGGAAGGCGGCTTCCTGGTCCACGGGTCCGCACCGGAGTCGCTGACCA<br>GCGCAGTCGAGAAGGCCGGAGGCCGCGTCGTGCCGGTCGCCTCGGCCGACCGCGAAG<br>CGCTCGCGGCGGCCCTGCGGGAGGTGCCGGGCGAGGTCGCCGGCGTGCTCTCGGTCC<br>ACACCGGCGCCGCAACGCACCTCGCCCTGCACCAGTCGCTGGGTGAGGCCGGCGTGC<br>GGGCCCCGCTCTGGCTGGTCACCAGCCGAGCGGTCGCGCTCGGGGAGTCCGAGCCGG<br>TCGATCCCGAGCAGGCGATGGTGTGGGGTCTCGGGCGCGTCATGGGCCTGGAGACCC<br>CGGAACGGTGGGGCGGTCTGGTGGACCTGCCCGCCGAACCCGCGCCGGGGGACGGCG<br>AGGCGTTCGTCGCCTGCCTCGGCGCGGACGGCCACGAGGACCAGGTCGCGATCCGTG<br>ACCACGCCCGCTACGGCCGCCGCCTCGTCCGCGCCCCGCTGGGCACCCGCGAGTCGA<br>GCTGGGAGCCGGCGGGCACGGCGCTGGTCACCGGCGGCACCGGTGCGCTCGGCGGCC<br>ACGTCGCCCGCCACCTCGCCAGGTGCGGGGTGGAGGACCTGGTGCTGGTCAGCAGGC<br>GCGGCGTCGACGCTCCCGGCGCGGCCGAGCTGGAAGCCGAACTGGTCGCCCTCGGCG<br>CGAAGACGACCATCACCGCCTGCGACGTGGCCGACCGCGAGCAGCTCTCCAAGCTGC<br>TGGAAGAACTGCGCGGGCAGGGACGTCCGGTGCGGACCGTCGTGCACACCGCCGGGG

	TGCCCGAATCGAGGCCGCTGCACGAGATCGGCGAGCTGGAGTCGGTCTGCGCGGCGA<br>AGGTGACCGGGGCCCGGCTGCTCGACGAGCTGTGCCCGGACGCCGAGACCTTCGTCC<br>TGTTCTCGTCCGGAGCGGGGGTGTGGGGCAGTGCGAACCTCGGCGCCTACTCCGCGG<br>CCAACGCCTACCTCGACGCGCTGGCCCACCGCCGCCGTGCGGAAGGCCGTGCGGCGA<br>CGTCCGTCGCGTGGGGCGCCTGGGCGGGCGAGGGCATGGCCACCGGCGACCTCGAGG<br>GGCTCACCCGGCGCGGCCTGCGCCCGATGGCGCCCGAGCGCGCGATCCGCGCGCTGC<br>ACCAGGCGCTGGACAACGGCGACACGTGCGTTTCGATCGCCGACGTCGACTGGGAGC<br>GCTTCGCGGTCGGCTTCACCGCCGCCCGGCCGCGTCCGCTGCTGGACGAGCTCGTCA<br>CGCCGGCGGTGGGGGCCGTCCCCGCGGTGCAGGCGGCCCCGGCGCGGGAGATGACGT<br>CGCAGGAGTTGCTGGAGTTCACGCACTCGCACGTCGCGGCGATCCTCGGGCATTCCA<br>GCCCGGACGCGGTCGGGCAGGACCAGCCGTTCACCGAGCTCGGCTTCGACTCGCTGA<br>CCGCGGTCGGGCTGCGCAACCAGCTCCAGCAGGCCACCGGGCTCGCGCTGCCCGCGA<br>CCCTGGTGTTCGAGCACCCCACGGTCCGCAGGTTGGCCGACCACATAGGACAGCAGC<br>TCGACAGCGGGACTCCCGCCCGGGAAGCGAGCAGCGCTCTTCGCGACGGCTACCGGC<br>AGGCGGGCGTGTCGGGCAGGGTCCGGTCCTACCTCGACCTGCTGGCGGGGCTGTCGG<br>ACTTCCGCGAGCACTTCGACGGCTCCGACGGGTTCTCCCTCGATCTCGTGGACATGG<br>CCGACGGTCCCGGAGAGGTCACGGTGATCTGCTGCGCGGGAACGGCGGCGATCTCCG<br>GTCCGCACGAGTTCACCCGGCTCGCCGGGGCGCTGCGCGGAATCGCTCCGGTTCGGG<br>CCGTGCCCCAGCCCGGCTACGAGGAGGGCGAACCTCTGCCGTCGTCGATGGCGGCGG<br>TGGCGGCGGTGCAGGCCGATGCGGTCATCAGGACACAGGGGGACAAGCCGTTCGTGG<br>TGGCCGGTCACTCCGCGGGGGCACTGATGGCCTACGCGCTGGCGACCGAACTGCTCG<br>ATCGCGGGCACCCGCCACGCGGTGTCGTCCTGATCGACGTCTACCCGCCCGGTCACC<br>AGGACGCGATGAACGCCTGGCTGGAGGAGCTGACCGCCACGCTGTTCGACCGCGAGA<br>CGGTGCGGATGGACGACACCAGGCTCACCGCCCTGGGCGCCTACGACCGCCTCACCG<br>GTCAGTGGCGACCCCGGGAAACCGGGCTGCCGACGCTGCTGGTCAGCGCCGGCGAGC<br>CGATGGGTCCGTGGCCCGACGACAGCTGGAAGCCGACGTGGCCCTTCGAGCACGACA<br>CCGTCGCCGTCCCCGGCGACCACTTCACGATGGTGCAGGAACACGCCGACGCGATCG<br>CGCGGCACATCGACGCCTGGCTGGGCGGAGGGAATTCAAGA
**Ery6(EpoAT4)TE**<br>Highlighted in red is the AT6 sequence.<br>Highlighted in	ATGACGAGTTCCAACGAACAGTTGGTGGACGCTCTGCGCGCCTCTCTCAAGGAGAAC<br>GAAGAACTCCGGAAAGAGAGCCGTCGCCGGGCCGACCGTCGGCAGGAGGAGATCGCG<br>ATCGTCGGCATGGCCTGCCGCTTCCCCGGCGGCGTGCACAACCCCGGTGAGCTGTGG<br>GAGTTCATCGTCGGCGGCGGAGACGCCGTGACGGAGATGCCCACCGACCGCGGCTGG<br>GACCTCGACGCGCTGTTCGACCCCGACCCGCAGCGCCACGGAACCAGCTACTCGCGA<br>CACGGCGCGTTCCTCGACGGGGCCGCCGACTTCGACGCGGCGTTCTTCGGGATCTCG

**blue** is the small subunit motif.	CCGCGCGAGGCGCTGGCGATGGACCCGCAGCAGCGCCAGGTCCTGGAAACGACGTGG<br>GAGCTGTTCGAGAACGCCGGCATCGACCCGCACTCGCTGCGGGGCAGCGACACCGGC<br>GTCTTCCTCGGCGCCGCGTACCAGGGCTACGGCCAGGACGCGGTGGTGCCCGAGGAC<br>AGCGAGGGCTACCTGCTCACCGGCAACTCCTCCGCCGTGGTGTCCGGCCGGGTCGCC<br>TACGTGCTGGGGCTGGAAGGCCCCGCGGTCACGGTGGACACGGCGTGTTCGTCGTCG<br>TTGGTGGCCTTGCATTCGGCGTGTGGGTCGTTGCGTGACGGTGACTGCGGTCTTGCG<br>GTGGCCGGTGGTGTGTCGGTGATGGCGGGCCCGGAGGTGTTCACCGAGTTCTCCCGC<br>CAGGGCGGCTTGGCCGTGGACGGGCGCTGCAAGGCGTTCTCCGCGGAGGCCGACGGC<br>TTCGGTTTCGCCGAGGGCGTCGCGGTGGTCCTGCTCCAGCGGTTGTCCGACGCCCGC<br>AGGGCGGGTCGCCAGGTGCTCGGCGTGGTCGCGGGCTCGGCGATCAACCAGGACGGC<br>GCGAGCAACGGTCTCGCGGCGCCGAGCGGCGTCGCCCAGCAGCGCGTGATCCGCAAG<br>GCGTGGGCGCGTGCGGGGATCACGGGCGCGGATGTGGCCGTGGTGGAGGCGCATGGG<br>ACCGGTACGCGGCTGGGCGATCCGGTGGAGGCGTCGGCGTTGCTGGCTACTTACGGC<br>AAGTCGCGCGGGTCGTCGGGCCCGGTGCTGCTGGGTTCGGTGAAGTCGAACATCGGT<br>CACGCGCAGGCGGCCGCGGGTGTCGCGGGCGTGATCAAGGTGGTCCTGGGGTTGAAC<br>CGCGGCCTGGTGCCGCCGATGCTCTGCCGCGGCGAGCGGTCGCCGCTGATCGAATGG<br>TCCTCGGGTGGTGTGGAACTTGCCGAGGCCGTGAGCCCGTGGCCTCCGGCCGCGGAC<br>GGGGTGCGCCGGGCCGGTGTGTCGGCGTTCGGGGTGAGCGGGACGAATGCACATGTT<br>ATCCTTGAGGAAGCCCCACCAGAAGAGGCAGCTGCAGCCGAGACTCCTGCCGAGGGT<br>ACTGGGGCCGTAGTACCTTGGGTCGTCTCGGGTCGTGGGGAAGAGGCTCTCAGAGCC<br>CAAGCCGCCCAACTTGCTGAGCACGTTCGTGACGACGACCAACGGCCAGCTTCCCCT<br>CTTGAGGTAGGTTGGTCACTTGCTACGACGCGTTCGGTTTTCGAGAATCGAGCAGTA<br>GTCGTTGGTGACGACCGGGACGCTTTGCTTGACGGGCTTCGGTCGTTGGCAGCTGGT<br>GAGGCTAGTCCTGACGTTGTAAGTGGGGCCGTCGGACCAACAGGACCTGGTCCCGTT<br>ATGGTCTTCCCAGGACAAGGCGGTCAATGGGTTGGTATGGGAGCCCGTCTTTTGGAC<br>GAGTCACCTGTTTTCGCCGCCAGAATCGCTGAGTGTGAGCAAGCATTAAGTGCATAC<br>GTTGACTGGAGTCTTACGGACGTTTTACGTGGAGACGGTAGTGAGTTGGCTAGAATA<br>GACGTAGTTCAACCCGTCTTGTGGGCCGTTATGGTCGCTTTAGCTGCCGTCTGGGCT<br>GACCAAGGTATCGAGCCCGCAGCCGTTGTAGGGCACTCTCAAGGTGAGATCGCCGCC<br>GCATGTGTTGTTGGAGCCATCTCACTTGACGAGGCAGCCCGTATCGTCGCAGTACGG<br>TCCGTATTGTTACGTCAACTTTCGGGTCGTGGTGGGATGGCATCATTAGGTATGGGT<br>CAAGAGCAAGCCGCAGACTTGATCGACGGACACCCTGGTGTTGTTGTTGCAGCCGTC<br>AATGGTCCTAGTTCTACAGTAATATCTGGGCCGCCTGAGGGAATAGCTGCCGTCGTC<br>GCCGACGCTCAAGAGCGAGGTTTGCGGGCCAGAGCTGTAGCATCAGACGTTGCTGGT

	CACGGTCCTCAATTGGACGCCATCCTTGACCAATTAACTGAGGGTTTGGCAGGGATA<br>CGTCCCGCTGCTACGGACGTAGCTTTCTACTCCACGGTAACAGCTGGGCACCTTACA<br>GACACAACAGAGCTTGACACGGCTTACTGGGTAAGAAATGTTCGTCGTACTGTTCGT<br>TTCGCCGACACTATAGACGCATTATTGGCAGACGGTTACCGTCTTTTCATCGAGGTA<br>TCCCCTCACCCTGTTCTCAATCTTGCTTTAGAGGGTTTAATCGAGCGTGCAGCTGTT<br>CCTGCCACTGTTGTCCCAACATTACGGAGAGACCACGGTGACACTACACAATTAGCC<br>CGAGCCGCAGCTCACGCTTTCGCTGCTGGTGCCGACGTTGACTGGCGGCGGTGGTTC<br>CCTGCTGATCCAGCACCTAGAACTGTAGACCGCTGGCGGGCAAGGGCGGCATGGTCT<br>CGTTGGCGGCTCCCGGTGAACGCGCCCGCGCGCTGATCGCACCGTGGGAGGACCGGA<br>TCTCCGTCGCGGCGGTCAACTCCCCGTCCTCGGTCGTGGTCTCCGGCGATCCGGAGG<br>CGCTGGCCGAACTCGTCGCACGTTGCGAGGACGAGGGCGTGCGCGCCAAG**ACGCTCC**<br>**CGGTGGACTACGCCTCGCAC**TCCCGCCACGTCGAGGAGATCCGCGAGACGATCCTCG<br>CCGACCTCGACGGCATCTCCGCGCGGCGTGCCGCCATCCCGCTCTACTCCACGCTGC<br>ACGGCGAACGGCGCGACGGCGCCGACATGGGTCCGCGGTACTGGTACGACAACCTGC<br>GCTCCCAGGTGCGCTTCGACGAGGCGGTCTCGGCCGCCGTCGCCGACGGTCACGCCA<br>CCTTCGTCGAGATGAGCCCGCACCCGGTGCTCACCGCGGCGGTGCAGGAGATCGCCG<br>CGGACGCCGTGGCCATCGGGTCGCTGCACCGCGACACCGCGGAGGAGCACCTGATCG<br>CCGAGCTCGCCCGGGCGCACGTGCACGGCGTGGCCGTGGACTGGCGGAACGTCTTCC<br>CGGCGGCACCTCCCGGTGGCGTGCCCAACTACCCGTTCGAGCCCCAGCGGTACTGGC<br>TCGCGCCGGAGGTGTCCGACCAGCTCGCCGACAGCCGCTACCGCGTCGACTGGCGAC<br>CGCTGGCCACCACGCCGGTGGACCTGGAAGGCGGCTTCCTGGTCCACGGGTCCGCAC<br>CGGAGTCGCTGACCAGCGCAGTCGAGAAGGCCGGAGGCCGCGTCGTGCCGGTCGCCT<br>CGGCCGACCGCGAAGCGCTCGCGGCGGCCCTGCGGGAGGTGCCGGGCGAGGTCGCCG<br>GCGTGCTCTCGGTCCACACCGGCGCCGCAACGCACCTCGCCCTGCACCAGTCGCTGG<br>GTGAGGCCGGCGTGCGGGCCCCGCTCTGGCTGGTCACCAGCCGAGCGGTCGCGCTCG<br>GGGAGTCCGAGCCGGTCGATCCCGAGCAGGCGATGGTGTGGGGTCTCGGGCGCGTCA<br>TGGGCCTGGAGACCCCGGAACGGTGGGGCGGTCTGGTGGACCTGCCCGCCGAACCCG<br>CGCCGGGGGACGGCGAGGCGTTCGTCGCCTGCCTCGGCGCGGACGGCCACGAGGACC<br>AGGTCGCGATCCGTGACCACGCCCGCTACGGCCGCCGCCTCGTCCGCGCCCCGCTGG<br>GCACCCGCGAGTCGAGCTGGGAGCCGGCGGGCACGGCGCTGGTCACCGGCGGCACCG<br>GTGCGCTCGGCGGCCACGTCGCCCGCCACCTCGCCAGGTGCGGGGTGGAGGACCTGG<br>TGCTGGTCAGCAGGCGCGGCGTCGACGCTCCCGGCGCGGCCGAGCTGGAAGCCGAAC<br>TGGTCGCCCTCGGCGCGAAGACGACCATCACCGCCTGCGACGTGGCCGACCGCGAGC<br>AGCTCTCCAAGCTGCTGGAAGAACTGCGCGGGCAGGGACGTCCGGTGCGGACCGTCG

	TGCACACCGCCGGGGTGCCCGAATCGAGGCCGCTGCACGAGATCGGCGAGCTGGAGT<br>CGGTCTGCGCGGCGAAGGTGACCGGGGCCCGGCTGCTCGACGAGCTGTGCCCGGACG<br>CCGAGACCTTCGTCCTGTTCTCGTCCGGAGCGGGGGTGTGGGGCAGTGCGAACCTCG<br>GCGCCTACTCCGCGGCCAACGCCTACCTCGACGCGCTGGCCCACCGCCGCCGTGCGG<br>AAGGCCGTGCGGCGACGTCCGTCGCGTGGGGCGCCTGGGCGGGCGAGGGCATGGCCA<br>CCGGCGACCTCGAGGGGCTCACCCGGCGCGGCCTGCGCCCGATGGCGCCCGAGCGCG<br>CGATCCGCGCGCTGCACCAGGCGCTGGACAACGGCGACACGTGCGTTTCGATCGCCG<br>ACGTCGACTGGGAGCGCTTCGCGGTCGGCTTCACCGCCGCCCGGCCGCGTCCGCTGC<br>TGGACGAGCTCGTCACGCCGGCGGTGGGGGCCGTCCCCGCGGTGCAGGCGGCCCCGG<br>CGCGGGAGATGACGTCGCAGGAGTTGCTGGAGTTCACGCACTCGCACGTCGCGGCGA<br>TCCTCGGGCATTCCAGCCCGGACGCGGTCGGGCAGGACCAGCCGTTCACCGAGCTCG<br>GCTTCGACTCGCTGACCGCGGTCGGGCTGCGCAACCAGCTCCAGCAGGCCACCGGGC<br>TCGCGCTGCCCGCGACCCTGGTGTTCGAGCACCCCACGGTCCGCAGGTTGGCCGACC<br>ACATAGGACAGCAGCTCGACAGCGGGACTCCCGCCCGGGAAGCGAGCAGCGCTCTTC<br>GCGACGGCTACCGGCAGGCGGGCGTGTCGGGCAGGGTCCGGTCCTACCTCGACCTGC<br>TGGCGGGGCTGTCGGACTTCCGCGAGCACTTCGACGGCTCCGACGGGTTCTCCCTCG<br>ATCTCGTGGACATGGCCGACGGTCCCGGAGAGGTCACGGTGATCTGCTGCGCGGGAA<br>CGGCGGCGATCTCCGGTCCGCACGAGTTCACCCGGCTCGCCGGGGCGCTGCGCGGAA<br>TCGCTCCGGTTCGGGCCGTGCCCCAGCCCGGCTACGAGGAGGGCGAACCTCTGCCGT<br>CGTCGATGGCGGCGGTGGCGGCGGTGCAGGCCGATGCGGTCATCAGGACACAGGGGG<br>ACAAGCCGTTCGTGGTGGCCGGTCACTCCGCGGGGGCACTGATGGCCTACGCGCTGG<br>CGACCGAACTGCTCGATCGCGGGCACCCGCCACGCGGTGTCGTCCTGATCGACGTCT<br>ACCCGCCCGGTCACCAGGACGCGATGAACGCCTGGCTGGAGGAGCTGACCGCCACGC<br>TGTTCGACCGCGAGACGGTGCGGATGGACGACACCAGGCTCACCGCCCTGGGCGCCT<br>ACGACCGCCTCACCGGTCAGTGGCGACCCCGGGAAACCGGGCTGCCGACGCTGCTGG<br>TCAGCGCCGGCGAGCCGATGGGTCCGTGGCCCGACGACAGCTGGAAGCCGACGTGGC<br>CCTTCGAGCACGACACCGTCGCCGTCCCCGGCGACCACTTCACGATGGTGCAGGAAC<br>ACGCCGACGCGATCGCGCGGCACATCGACGCCTGGCTGGGCGGAGGGAATTCAAGA

Supplementary Table S2. Low-resolution LC-MS retention times, calculated masses, and observed masses for Ery6TE and DEBS3-catalyzed reaction products. Samples were analyzed according to the method outlined in the **Supplemental Methods**. All samples were compared to the boiled control to confirm peak identity.

Compound	Retention Time (min)	Calculated Mass [M+H$^+$]	Observed Mass [M+H$^+$]	Δ Mass (Calc. – Obs.)
**4a**	3.9	155.1	155.1	0
**4b**	4.3	169.1	169.1	0
**4c**	4.5	183.1	183.1	0
**4d**	4.3	193.1	193.1	0
**4e**	4.9	197.1	197.1	0
**4f**	5.2	211.1	211.1	0
**6a**	4.38	297.2	297.2	0
**6f**	7.42	339.3	339.2	0.1
**7a**	N.D.	295.2	N.D.	-
**7f**	6.52	337.2	337.2	0
**8a**	7.45	355.2	355.2	0
**8b**	9.24	397.3	397.3	0
**8c**	10.49	439.3	439.3	0
**9a**	5.92	353.2	353.2	0
**9b**	9.22	395.3	395.3	0
**9c**	10.22	437.3	437.3	0

Supplementary Table S3. Low-resolution peak areas for motif-exchanged Ery6TE chimera reaction products with competing extender units. Extracted ion count (EIC) peak areas from one replicate are shown for each reaction condition. Mass counts <5,000 are shown as N.D. (Not Detected). Fold-increase relative to wild-type EryAT6 is shown in brackets and was calculated from the average peak area.

Entry	Enzyme	Representative EIC peak area for each product pyrone					
		**4a**	**4b**	**4c**	**4d**	**4e**	**4f**
1	EryAT6	559,712	272,9822	861,092	56,774	N.D.	95,864
2	EryAT6-$LS_{ThaAT13}$	10,046	7,920	N.D.	N.D.	49,992	250,937 (2.7)
3	EryAT6-L_{ThAT13}	8,179	68,166	N.D.	N.D.	N.D.	N.D.
4	EryAT6-$S_{ThaAT13}$	N.D.	N.D.	N.D.	N.D.	N.D.	18,500 (0.2)
5	EryAT6-LS_{CinAT1}	N.D.	N.D.	N.D.	N.D.	N.D.	N.D.
6	EryAT6-L_{CinAT1}	N.D.	12,440	N.D.	N.D.	N.D.	22,467 (0.3)
7	EryAT6-S_{CinAT1}	8,687	10,158	16,659	N.D.	10,582	96,446 (1.1)

Supplementary Table S4. CASTp 3.0 active site surface area and volume calculations of EryAT6 domain/motif-swapped variants. The percentage of **4f** production is from **Figure 3**.

Variant	Surface Area (Å^2)	Volume (Å^3)	Number of residues in pocket from LSM	Number of residues in pocket from SSM	% Fraction of 4f
EryAT6	507	461	1	6	1.9
Ery(AT6LS$_{CinAT1}$)TE	748	735	4	8	N.D.
Ery(AT6L$_{CinAT1}$)TE	479	387	1	6	64.2
Ery(AT6S$_{CinAT1}$)TE	839	807	3	8	70.2
Ery(AT6LS$_{ThaAT13}$)TE	596	573	3	8	78.2
Ery(AT6L$_{ThaAT13}$)TE	540	453	1	6	N.D.
Ery(AT6S$_{ThaAT13}$)TE	666	622	3	8	89.0
MonAT5LSCinAT1	782	663	4	8	9.5
MonAT5LCinAT1	829	777	4	8	51.5
MonAT5SCinAT1	765	692	3	7	33.0
EpoAT4	352	238	2	7	N.D.
EpoAT4LSThaAT13	570	365	4	9	10.0
EpoAT4LThaAT13	534	364	3	8	2.4
EpoAT4SThaAT13	625	399	4	9	32.5

Supplementary Table S5. Low-resolution peak areas for Ery6TE single mutant enzyme reaction products with competing extender units. Extracted ion count (EIC) peak areas from one replicate are shown for each reaction condition. Mass counts <5,000 are shown as N.D. (Not Detected).

Entry	Enzyme	Representative EIC peak area for each product pyrone					
		**4a**	**4b**	**4c**	**4d**	**4e**	**4f**
1	EryAT6	1,797,030	6,262,131	937,019	774,518	148,723	182,870
2	V612G	1,325,011	3,905,977	510,020	352,395	177,334	120,207
3	V612L	1,285,767	3,322,016	381,711	210,852	180,807	117,350
4	V615N	747,715	2,423,680	131,220	36,170	181,790	93,827
5	V615Q	1,097,426	2,738,643	N.D.	50,250	268,812	161,433
6	T739A	2,356,138	6,940,536	994,124	491,678	191,861	252,680
7	T739V	2,431,384	8,362,796	1,359,950	557,098	164,231	203,488
8	P741R	268,974	967,157	585,638	219,877	42,438	56,919
9	D743E	218,759	821,565	170,127	16,923	7,004	N.D.
10	Y744R	63,916	120,882	92,085	180,450	143,976	146,252
11	S746G	1,491,171	4,862,856	1,109,352	294,007	60,660	39,473

Supplementary Table S6. Low-resolution peak areas of Ery6TE double and triple mutant enzyme-catalyzed reaction products with competing extender units. Extracted ion count (EIC) peak areas from one replicate are shown for each reaction condition. Fold-increase relative to wild-type EryAT6 is shown in brackets and was calculated from the average peak area. Mass counts <5,000 are shown as N.D. (Not Detected).

Entry	Enzyme	Representative EIC peak area for each product pyrone					
		**4a**	**4b**	**4c**	**4d**	**4e**	**4f**
WT	EryAT6	1,797,030	6,262,131	937,019	774,518	148,723	182,870
1	T739A/P741R	40,288	299,105	59,747	18,575	N.D.	N.D.
2	T739A/D743E	35,201	352,161	25,127	5,862	N.D.	N.D.
3	T739A/Y744R	32,603	41,261	43,861	9,920	32,135	81,615 (0.5)
4	T739A/S746G	64,637	909,018	56,365	6,089	N.D.	N.D.
5	P741R/D743E	7,888	71,239	16,999	N.D.	N.D.	N.D.
6	P741R /P744R	N.D.	8,103	9,919	N.D.	N.D.	14,290 (0.09)
7	P741R/S746G	N.D.	35,658	N.D.	N.D.	N.D.	N.D.
8	D743E/P744R	50,150	114,906	82,897	15,423	96,913	299,030 (1.8)
9	D743E/S746G	223,077	715,543	75,108	6,700	5,769	9,128 (0.06)
10	Y744R/S746G	49,327	67,939	273,810	52,145	493,665	1,098,936 (6.5)
11	T739A/P741R/D743E	26,825	1,070,549	96,709	109,513	6,601	N.D.
12	T739A/P741R/Y744R	11,242	97,375	118,461	21,104	7,534	135,354 (0.9)
13	T739A/P741R/S746G	399,974	1,201,207	219,544	13,532	N.D.	N.D.
14	T739A/D743E/Y744R	29,515	19,904	220,767	N.D.	49,597	N.D.
15	T739A/Y744R/S746G	11,073	14,628	128,771	30,676	41,055	555,452 (3.3)
16	T739A/D743E/S746G	356,290	464,846	297,090	N.D.	65,237	N.D.
17	P741R/D743E/Y744R	58,687	39,439	380,885	9,105	56,165	12,988 (0.09)
18	P741R/D743E/S746G	86,384	1,705,683	34,766	18,578	N.D.	6,658 (0.04)
19	P741R/Y744R/S746G	10,566	16,069	184,080	24,747	19,765	346,789 (1.8)
20	D743E/Y744R/S746G	61,696	25,716	187,533	N.D.	132,376	280,781 (1.7)

Supplementary Table S7. Low-resolution peak areas of AT domain/motif-exchanged Ery6TE variant-catalyzed reaction products with competing extender units. Extracted ion count (EIC) peak areas from one replicate are shown for each reaction condition. Mass counts <5,000 are shown as N.D. (Not Detected). Fold-increase relative to the corresponding AT-swap is shown in brackets and was calculated from the average peak area, using the minimum detection limit of 5,000 mass ions where necessary.

Entry	Enzyme	Representative EIC peak area for each product pyrone					
		**4a**	**4b**	**4c**	**4d**	**4e**	**4f**
1	EryAT6	97,320	467,447	248,294	14,649	7,329	17,126
2	MonAT5	33,112	156,981	63,487	N.D.	N.D.	N.D.
3	MonAT5-LS_{CinAT1}	32,024	26,044	N.D.	N.D.	N.D.	6,908 (1.3)
4	MonAT5-L_{CinAT1}	7,886	13,531	58,701	N.D.	15,936 (3.2)	101,559 (20)
5	MonAT5-S_{CinAT1}	24,241	16,660	19,633	N.D.	6,584	33,371 (7)
6	EpoAT4	44,645	35,480	N.D.	N.D.	N.D.	N.D.
7	EpoAT4-$LS_{ThaAT13}$	17,633	8,294	45,078 (9)	17,904 (3.6)	N.D.	8,229 (1.6)
8	EpoAT4-$L_{ThaAT13}$	69,563	30,385	26,727 (5.3)	N.D.	N.D.	9,870 (2)
9	EpoAT4-$S_{ThaAT13}$	32,577	15,952	36,864 (7.4)	N.D.	N.D.	39,588 (8)

Supplementary Table S8. *E. coli* strains used in this study.

**Bacterial Strain**	**Genotype/ Description**	**Purpose**	**Reference**
E. coli TOP10	F- mcrA Δ(mrr-hsdRMS-mcrBC) φ80lacZΔM15 ΔlacX74 recA1 araD139 Δ(ara-leu)7697 galU galK λ- rpsL(StrR) endA1 nupG	Cloning and DNA manipulation	Invitrogen
E. coli BL21 (DE3)	F¯ ompT gal dcm lon hsdSB(rB- mB-) λ(DE3 *lacI lacUV5-T7 gene 1 ind1 sam7 nin5]	Expression of MatB	Invitrogen
E. coli K207-3	F^- *ompT hsdS* (r_B^- m_B^-) *gal dcm* (DE3) *panD*::*panDS2SA*D*prpRBCD*::T7 prom-*sfp* T7 prom-*prpE ygfG*::T7 prom-*accA1*-T7 prom-*pccB* DTolC	Expression of PKS modules	Murli, S.; Kennedy, J.; Dayem, L. C.; Carney, J. R.; Kealey, J. T. Metabolic Engineering of Escherichia Coli for Improved 6-Deoxyerythronolide B Production. *J Industrial Microbiol Biotechnology* **2003**, *30* (8), 500–509

Supplementary Table S9. Oligonucleotides used in this study. Mutations are in red lowercase while the annealing section of the primer is black uppercase. Forward mutation primers paired with Gibson.F to amplify half of the Ery6TE plasmid backbone and vice versa with reverse set.

**Primer Name**	**5' ->3' Primer Sequence**	**Primer Function**
V612A.F	ACGTCcgcCCGCTCCAGCGACG	Ery6TE mutation
V612A.R	AGCGGgcgGACGTCGTACAGCC	Ery6TE mutation
V615A.F	ACCGGCTGcgcGACGTCGACC	Ery6TE mutation
V615A.R	ACGTCgcgCAGCCGGTGTTGTTCTCC	Ery6TE mutation
T739A.F	gccCTCCCGGTGGACTACGC	Ery6TE mutation
T739A.R	CTTGGCGCGCACGC	Ery6TE mutation
P741A.F	TCCACggcGAGCGTCTTGGCGC	Ery6TE mutation
P741A.R	ACGCTCgccGTGGACTACGCC	Ery6TE mutation
V612G.F	ggcGACGTCGTACAGCCGGTGTTGTTCTCC	Ery6TE mutation
V612L.F	ctgGACGTCGTACAGCCGGTGTTGTTCTCC	Ery6TE mutation
V612G/L.R	CCGCTCCAGCGACGGCG	Ery6TE mutation
V615N.F	aacCAGCCGGTGTTGTTCTCC	Ery6TE mutation
V615Q.F	cagCAGCCGGTGTTGTTCTCC	Ery6TE mutation

V615N/Q.R	GACGTCGACCCGCTCC	Ery6TE mutation
T739A.F	gccCTCCCGGTGGACTACGC	Ery6TE mutation
T739V.F	gtcCTCCCGGTGGACTACGC	Ery6TE mutation
T739A/V.R	CTTGGCGCGCACGC	Ery6TE mutation
P741R.F	ACGCTCcgcGTGGACTACGCCTCGCACTCCCG	Ery6TE mutation
P741R.R	TCCACgcgGAGCGTCTTGGCGCGCACGCCC	Ery6TE mutation
Y744R.F	tacGCCTCGCACTCCCG	Ery6TE mutation
Y744R.R	GTCCACCGGGAGCG	Ery6TE mutation
S746G.F	ggcCACTCCCGCCACGTC	Ery6TE mutation
S746G.R	GGCGTAGTCCACCGGG	Ery6TE mutation
V615Q/E.F	TCGACGTCnagCAGCCGGTGTTGTTCTCCGTGATGGTGTCGC	Ery6TE mutation
V615Q/E.R	ACCGGCTGctnGACGTCGACCCGCTCCAGC	Ery6TE mutation
V615C/W.R	ACCGGCTGncaGACGTCGACCCGCTCCAGC	Ery6TE mutation
V615C/W.F	TCGACGTCtgnCAGCCGGTGTTGTTCTCCGTGATGGTGTCGC	Ery6TE mutation
V615H.F	TCGACGTCcacCAGCCGGTGTTGTTCTCCGTGATGGTGTCGC	Ery6TE mutation
V615H.R	ACCGGCTGgtgGACGTCGACCCGCTCCAGC	Ery6TE mutation

V615X.F	TCGACGTCnnkCAGCCGGTGTTGTTCTCCGTGATGGTGTCGC	Ery6TE mutation
V615X.R	ACCGGCTGmnnGACGTCGACCCGCTCCAGC	Ery6TE mutation
Gibson.F	TGCGCCGGTTGCATTCGATTCC	Ery6TE mutation
Gibson.R	AGACTTGTTCAACAGGCCAGCCATTACG	Ery6TE mutation
MonAT5_ATinsertGA.F	TGAGCCCGTGGCCTCCG	Motif swap into Ery6TE
MonAT5_ATinsertGA.R	TCGGACACCTCCGGCGC	Motif swap into Ery6TE
AT_GAinsertback.F	TACCCGTTCGAGCCCCAGC	Motif swap into Ery6TE
AT_GAinsertback.R	AACGCCGACACACCGGCC	Motif swap into Ery6TE
LGCinSwapMonAT5.F	tcagcagccgGTCTTGTGGGCCGTTATGG	Motif swap into Ery6TE
LGCinSwapMonAT5.R	atatcgccttcAGCCAACTCACTACCGTCTCC	Motif swap into Ery6TE

LGThaMonswap.F	GTGAATCAGCCGGTCTTGTGGGCCGTTATGG	Motif swap into Ery6TE
LGThaMonswap.R	atccagacgAGCCAACTCACTACCGTCTCC	Motif swap into Ery6TE
SmThaMonswap.R	ggcacccggcagaacTCTGGCCCGCAAACCTCG	Motif swap into Ery6TE
SmThaMonswap.F	GATGCCGCAGGCCACGGTCCTCAATTGGACGCCATCC	Motif swap into Ery6TE
SmCinMonswap.F	acgcgcaggccacGGTCCTCAATTGGACGCCATCC	Motif swap into Ery6TE
SmCinMonswap.R	tccacgcgcagggcTCTGGCCCGCAAACCTCG	Motif swap into Ery6TE
EpoAT4_ATinsertGA.F	TGAGCCCGTGGCCTCCG	EpoAT4 and motif swap into Ery6TE
EpoAT4_ATinsertGA.R	TCGGACACCTCCGGCGC	EpoAT4 and motif

		swap into Ery6TE
AT_GAinsertback.F	TACCCGTTCGAGCCCCAGC	EpoAT4 and motif swap into Ery6TE
AT_GAinsertback.R	AACGCCGACACACCGGCC	EpoAT4 and motif swap into Ery6TE
SmCinEposwap.F	acgcgcaggccacTCTCCATTGATGGAGCCTATG	EpoAT4 and motif swap into Ery6TE
SmCinEposwap.R	tccacgcgcagggcCTTCGTTCTTACACCTCTTGC	EpoAT4 and motif swap into Ery6TE
SmThaEposwap.F	ATGCCGCAGGCCACTCTCCATTGATGGAGCCTATGG	EpoAT4 and motif swap into Ery6TE

SmThaEposwap.R	ggcacccggcagaacCTTCGTTCTTACACCTCTTGC	EpoAT4 and motif swap into Ery6TE
LGThaEposwap.F	GTGAATCAGCCGGCCTTGTTCACTGTAGAG	EpoAT4 and motif swap into Ery6TE
LGThaEposwap.R	atccagacgGTCGAGAAGCAATGACTC	EpoAT4 and motif swap into Ery6TE
LgCinEposwap.F	tcagcagccgGCCTTGTTCACTGTAGAG	EpoAT4 and motif swap into Ery6TE
LgCinEposwap.R	atatcgccttcGTCGAGAAGCAATGACTC	EpoAT4 and motif swap into Ery6TE

Supplemental Figures

Supplementary Figure S1. SDS-Page analysis of clarified cellular lysates of expressed Ery6TE modules. M, Protein ladder; Lane 1, wild-type Ery6TE (10 µL); Lane 2, wild-type Ery6TE (5 µL); Lane 3, EryAT6-LS$_{CinAT1}$ CinAT1 (10 µL); Lane 4, EryAT6-LS$_{CinAT1}$ CinAT1 (10 µL). The approximate molecular weight of the Ery6TE module is 180 kDa.

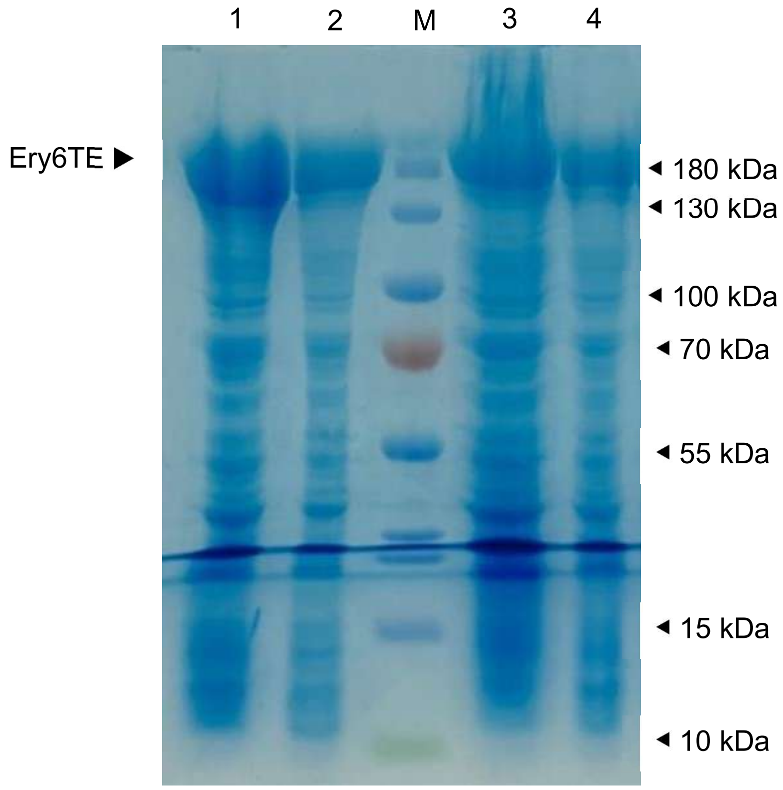

Supplementary Figure S2. Representative extracted ion chromatograms of Ery6TE-catalyzed reactions.

A

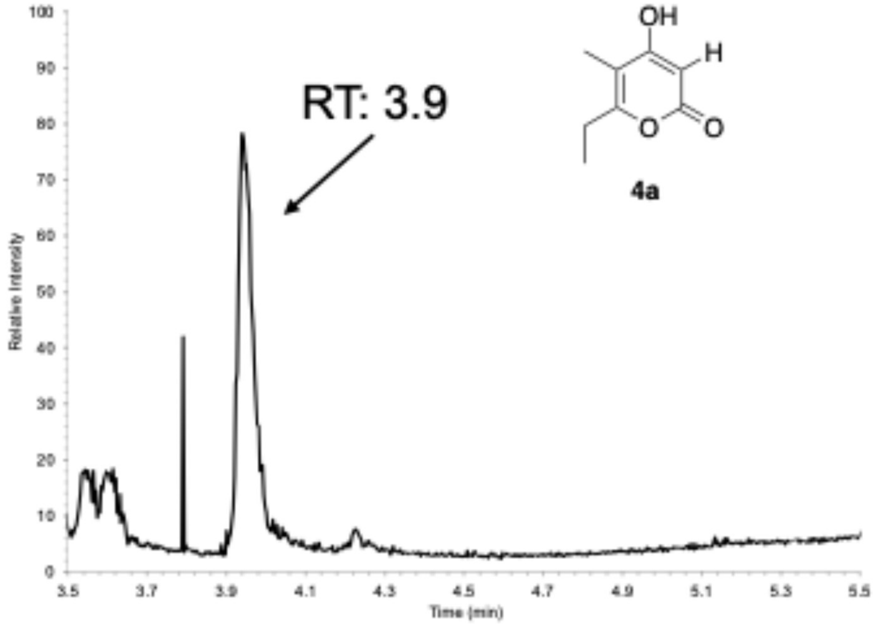

B

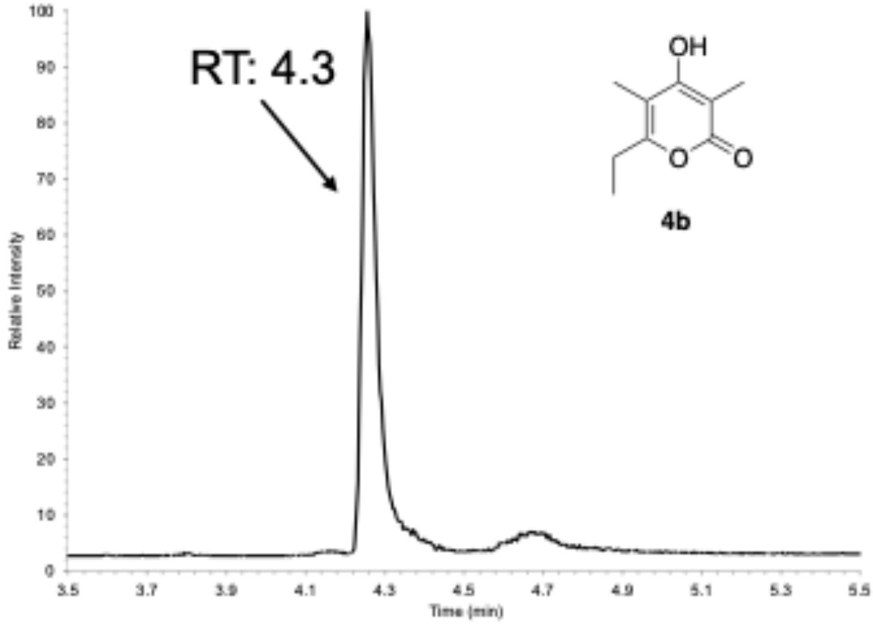

C

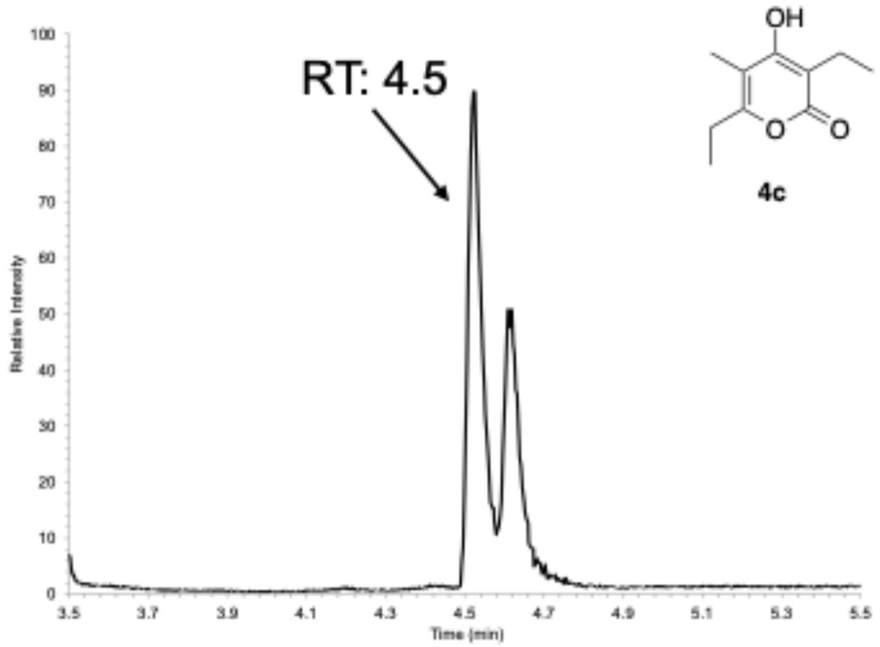
RT: 4.5
OH
O
O
4c
Relative Intensity
Time (min)

D

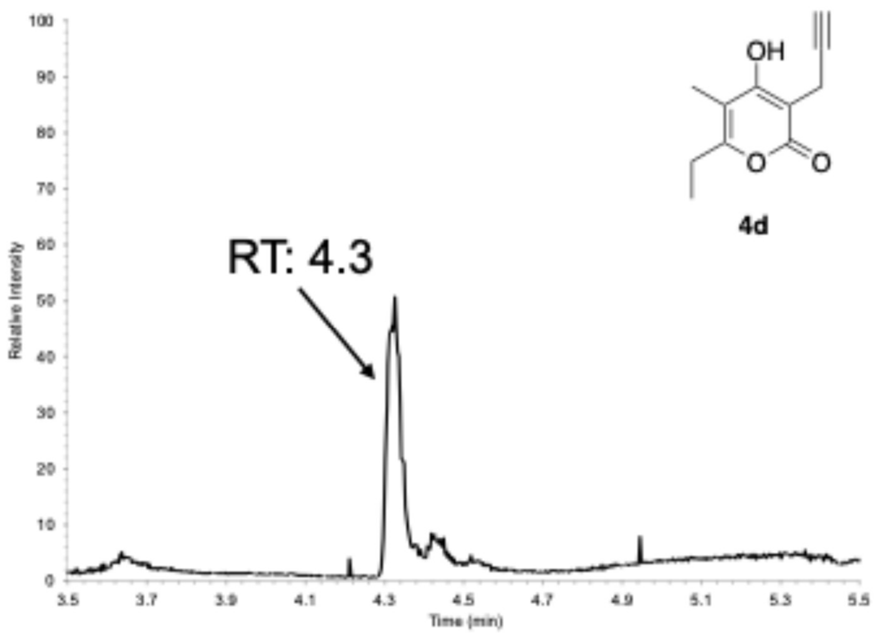
RT: 4.3
OH
O
O
4d
Relative Intensity
Time (min)

E

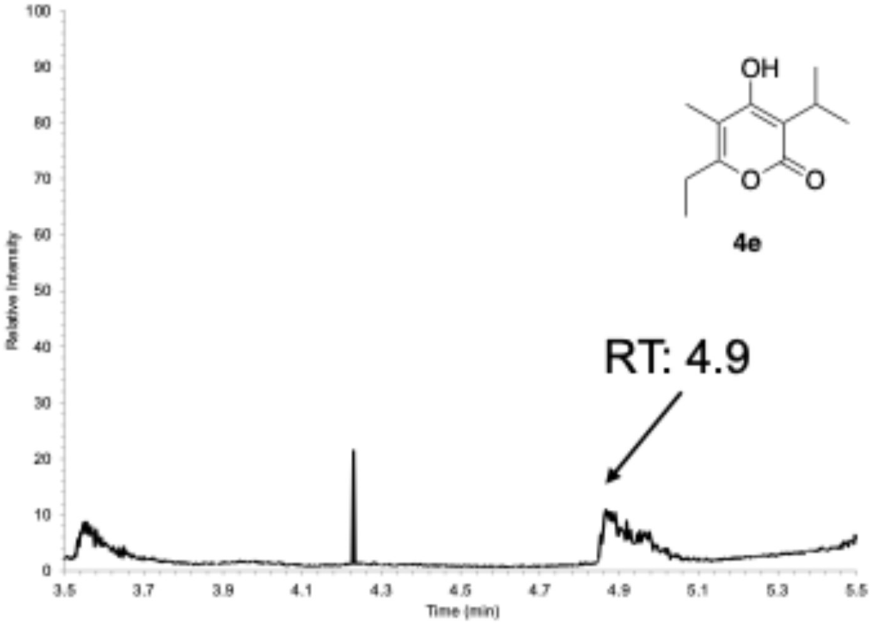

OH
O
O
4e
RT: 4.9
Relative Intensity
Time (min)

F

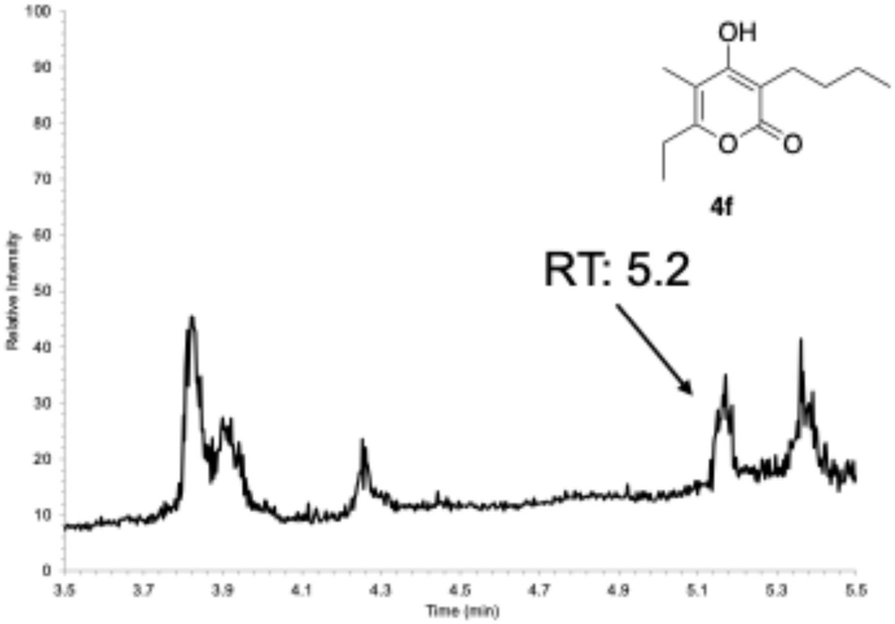

OH
O
O
4f
RT: 5.2
Relative Intensity
Time (min)

Supplemental Methods

Expression and Purification of WT and Mutant MatB Enzymes

Wild-type Ery6TE and mutant plasmids were transformed into chemically competent *E. coli* K207-3 (**Supplementary Table S8**). Colonies were grown on LB agar plates supplemented with 30 µL/mL Kan, and a single colony was selected for overnight incubation in 5mL LB broth supplemented with 30 µL/mL Kan. A 3 mL aliquot of the overnight culture was used to inoculate 300 mL of LB broth, which was incubated at 37 °C with shaking at 250 rpm until an optical density of 0.6 was reached. The culture was then induced by the addition of isopropyl beta-D-1-thiogalactopyranoside (IPTG) to 1 mM and incubated at 18 °C with shaking at 250 rpm for 20 h. Cells were then collected by centrifugation at 4,800 *g* for 15 min at 4 °C. The supernatant broth was decanted, and the resulting cell pellet was resuspended in a protein wash buffer containing 50 mM Tris-HCl, 300 mM NaCl, and 20 mM imidazole. Resuspended cells were lysed by sonication, and the cell lysate was isolated by centrifugation at 10,000 *g* and 4 °C for 1 h. The cell lysate was then purified by fast protein liquid chromatography (Profinia System, Bio-Rad) using the native IMAC system consisting of 7 wash solutions; 1 (wash 1) 600 mM KCl, 100 mM KH_2PO_4, 10 mM imidazole, pH 8.0, 2 (wash 2) 600 mM KCl, 100 mM, KH_2PO_4, 20 mM imidazole, pH 8.0, 3 (elution) 600 mM KCl, 100 mM KH_2PO_4, 500 mM imidazole, pH 8.0, 4 (desalting) 685 mM NaCl, 13.5 mM KCl, 21.5 mM Na_2HPO_4, 40.5 mM KH_2PO_4, pH 7.0, 5 (cleaning 1) 1 M NaCl, 100 mM Tris, pH 8.0, 6 (cleaning 2): 2 M NaCl, 400 mM NaOAc, pH 4.5, 7 (storage): 4% benzyl alcohol in water. Once purified, the protein was concentrated with Macrosep Advance 30K MWCO centrifugal filters (Pall Corp., Puerto Rico). The protein buffer was switched to protein storage buffer (50 mM Tris hydrochloride and 500 mM NaCl) through 2 centrifugation cycles. After the last centrifugation, glycerol was added to the protein storage buffer to a volume of 10%. The protein was then aliquoted and stored at -80 °C. The purity of the target protein was analyzed by SDS-PAGE and quantified using the Bradford protein assay kit supplied by Bio-Rad.

Construction of Ery6TE Single/Double/Triple Mutants

Phusion Hot Start II High-Fidelity DNA Polymerase (Thermo Fisher; Lithuania) was used to amplify two fragments of the PDD Ery6TE pET28a plasmid. Primers within EryAT6 were designed to introduce the amino acid replacement at the desired site. PCR reactions were then run on 1.0% agarose gels, and the desired bands were extracted using a gel extraction kit (Monarch BioLabs). Fragment DNA concentration was measured using a Nanodrop Lite Spectrophotometer (Fischer Scientific). Homologous regions at the ends of the DNA fragments were used for Gibson Assembly (NEB), and plasmids were constructed using the manufacturer's protocols. Resulting plasmids were transformed into chemically competent *E. coli*

Top10 grown on LB-Agar-Kan. After overnight growth at 37 °C, a single colony was picked to inoculate 5 mL of LB-Kan. Overnight cultures were miniprepped (New England BioLabs) to purify the plasmid. Mutations were verified with Sanger sequencing (Genewiz). To construct the panel of double and triple amino acid mutation swaps, one of the single amino acid swaps was used as a template for the PCR. The Gibson assembly protocol was then repeated. Once double amino acid swaps were sequence confirmed, the same process was repeated once more to create a third mutation.

Construction of Ery6TE Single Motif Swaps

Phusion Hot Start II High-Fidelity DNA Polymerase (Thermo Fisher; Lithuania) was used to amplify two fragments of the PDD Ery6TE pet28a plasmid backbone without the respective motif. Two separate primers were designed to amplify the desired motif from CinAT1 and ThaAT13 chimeras. PCR reactions were then run on 1.0% agarose gels, and the desired bands were extracted using a gel extraction kit (Monarch BioLabs). Fragment DNA concentration was measured using a Nanodrop Lite Spectrophotometer (Fischer Scientific). All amplified fragments were added to a Phusion PCR reaction in equimolar ratios. Resulting plasmids were transformed into chemically competent *E. coli* Top10 grown on LB-Agar-Kan. After overnight growth at 37 ˚C, a single colony was picked to inoculate 5 mL of LB-Kan. Overnight cultures were miniprepped (New England BioLabs) to purify the plasmid. Mutations were verified with Sanger sequencing (Genewiz). To construct the panel of double and triple amino acid mutation swaps, one of the single amino acid swaps was used as a template for the PCR. The Gibson assembly protocol was then repeated. Once double amino acid swaps were sequence confirmed, the same process was repeated once more to create a third mutation.

Construction of Ery6TE AT Swaps

Gene blocks were ordered from IDT (Coralville, IA) to match those previously described for MonAT5 and EpoAT4[78]. Gene blocks were ordered with 40-55bp of homology to Ery6TE WT_pet28a on each end of linear DNA. Primers were designed to amplify the gblock DNA and the Ery6TE WT_pet28a backbone so that each fragment possessed homologous ends. MonAT5_ATinsertGA.F and MonAT5_ATinsertGA.R were used to amplify the MonAT5 gblock insert while AT_GAinsertback.F and AT_GAinsertback.R amplified the Ery6TE WT_pet28a backbone. Phusion Hot Start II High-Fidelity DNA Polymerase (Thermo Fisher; Lithuania) was used to amplify both fragments. PCR reactions were then run on 1.0% agarose gels, and the desired bands were extracted using a gel extraction kit (Monarch BioLabs). Fragment DNA concentration was measured using a Nanodrop Lite Spectrophotometer (Fischer Scientific). Homologous regions at the ends of the DNA fragments were used for Gibson Assembly

(NEB), and plasmids were constructed using manufacturer protocols. Resulting plasmids were transformed into chemically competent *E. coli* Top10 grown on LB-Agar-Kan. After overnight growth at 37 °C, a single colony was picked to inoculate 5 mL of LB-Kan. Overnight cultures were miniprepped (New England BioLabs) to purify the plasmid. Mutations were verified with whole plasmid sequencing (Genewiz; South Plainfield, NJ).

Modeling of EryAT6 Motif Swaps and CASTp Analysis

3D models of EryAT6 motif swaps were generated using AlphaFold3.[1] The AT models were generated using the translated amino acid sequences from the nucleotide sequences in Supplemental Table S1. Each protein was subjected to five predicted models, and the model with the highest predicted quality was used in downstream analysis. CASTp 3.0 was used to generate catalytic pocket volumes.[2] The default radius probe of 1.4 angstroms was maintained for all analyses.

Supplemental References

(1) Abramson, J.; Adler, J.; Dunger, J.; Evans, R.; Green, T.; Pritzel, A.; Ronneberger, O.; Willmore, L.; Ballard, A. J.; Bambrick, J.; Bodenstein, S. W.; Evans, D. A.; Hung, C.-C.; O'Neill, M.; Reiman, D.; Tunyasuvunakool, K.; Wu, Z.; Žemgulytė, A.; Arvaniti, E.; Beattie, C.; Bertolli, O.; Bridgland, A.; Cherepanov, A.; Congreve, M.; Cowen-Rivers, A. I.; Cowie, A.; Figurnov, M.; Fuchs, F. B.; Gladman, H.; Jain, R.; Khan, Y. A.; Low, C. M. R.; Perlin, K.; Potapenko, A.; Savy, P.; Singh, S.; Stecula, A.; Thillaisundaram, A.; Tong, C.; Yakneen, S.; Zhong, E. D.; Zielinski, M.; Žídek, A.; Bapst, V.; Kohli, P.; Jaderberg, M.; Hassabis, D.; Jumper, J. M. Accurate Structure Prediction of Biomolecular Interactions with AlphaFold 3. *Nature* **2024**, *630* (8016), 493–500. https://doi.org/10.1038/s41586-024-07487-w.

(2) Tian, W.; Chen, C.; Lei, X.; Zhao, J.; Liang, J. CASTp 3.0: Computed Atlas of Surface Topography of Proteins. *Nucleic Acids Res.* **2018**, *46* (W1), W363–W367. https://doi.org/10.1093/nar/gky473.
